# Assessment of Local Strain of PVC-P Geomembranes Induced by Particle Cushion Layers

**DOI:** 10.3390/polym18172137

**Published:** 2026-09-02

**Authors:** Yunyun Wu, Kefan Jiao, Shengyi Hou, Pengpeng Yang, Zikuo Jia, Xianlei Zhang

**Affiliations:** 1 School of Water Conservancy, North China University of Water Resources and Electric Power, Zhengzhou 450046, China; wuyunyun01612@163.com (Y.W.);; 2 Jilin Baishan Hydropower Plant, Songhua River Hydropower Co., Ltd., Jilin 132404, China

**Keywords:** PVC-P geomembrane, cushion layer, void width–depth ratio, linear strain, areal strain, allometric function

## Abstract

This study investigates the deformation of plasticized polyvinyl chloride (PVC-P) geomembranes (GMBs) on uniform cushions and flat-rounded gravel cushions using a self-developed testing apparatus for GMB deformation and a three-dimensional (3D) laser scanner. Based on the principle of square voids equivalent to circles, the void width–depth ratio was adopted to quantify voids within granular cushions. The models between strain and void width–depth ratio, as well as gradation parameters, were established. The gradation parameters of flat-rounded gravel cushions satisfying the deformation requirements were determined for GMBs as anti-seepage materials in basins of pumped storage power stations. The results demonstrate that the areal strain of the PVC-P GMB is consistently greater than its linear strain for uniform cushions. From the perspective of engineering safety, the areal strain is recommended to characterize the deformation of PVC-P GMBs. The GMB areal strain on flat-rounded gravel cushions exhibits an allometric function with the width–depth ratio and non-uniformity coefficient, and an Exp2P function with the maximum limiting particle size, with all determination coefficients exceeding 0.99. Based on the areal strain less than 20% of its elastic strain threshold, the granular cushion should satisfy a non-uniformity coefficient in the range of 15.3 to 20.7, and the maximum limiting particle size is between 4.75 and 23.54 mm, the PVC-P GMB can maintain elastic deformation behavior, which extends its service life in practical engineering. These findings contribute to a deeper understanding of the local deformation behavior of PVC-P GMBs on cushion layers. The gradation parameters satisfying the deformation requirements for particle cushion layers provide technical guidance for cushion layer design for the application of PVC-P GMBs in pumped storage power stations.

## 1. Introduction

Pumped storage power stations are being constructed on a large scale to achieve the dual carbon goal in China, and the reservoirs must be lined with durable watertight facings to prevent water loss and ensure structural safety. Plasticized polyvinyl chloride (PVC-P) geomembranes (GMBs) are considered a strong candidate for the liner material in these applications due to their exceptional impermeability, outstanding flexibility, high puncture resistance, convenience for construction, and cost-effectiveness [1,2,3,4]. In engineering, a cushion layer is typically placed beneath the GMB to achieve water drainage. However, the gravel cushion layer may induce excessive tensile strains in the GMB, which may lead to GMB puncture and affect the safety of the project. Consequently, evaluation of the strains in GMBs induced by cushion layers is of significant importance.

The strains of a GMB are typically determined by index tests, or performance tests. Index tests provide a measure of strain caused by idealized gravels or subgrade [5,6], whereas performance tests provide strain induced by site-specific gravel and subgrade [7,8]. Bennett and Brachman [9] demonstrated that large discrepancies existed between results from index tests and performance tests, and recommended conducting performance tests using site-specific materials to investigate strain. Researchers typically placed a thin, deformable lead or aluminum sheet beneath the GMB to preserve its final deformed shape and then deduced local strains [10,11]. The deformed shape of the lead sheet was measured with a FaroArm three-dimensional (3D) laser scanner [9,12]. Several methods have been developed to calculate strains from the deformed shape of the GMB. The segment elongation method [5] calculated membrane strain assuming the GMB experienced only vertical displacement. Tognon et al. [7] considered both the membrane strain and the bending strains to evaluate the strains on the upper and lower surfaces of the high-density polyethylene (HDPE) GMBs. Both methods underestimated the strains since they neglected the horizontal displacements. Recently, Eldesouky and Brachman [12,13] presented a new thin plate method to calculate local strains of HDPE GMBs from the deformed shape imposed by coarse gravels. Brachman et al. [14] also adopted the thin plate method to calculate the strains considering creep effects, as the screening tests were conducted at 55 °C for 100 h to accelerate creep. The above studies were regarding HDPE strains from overlying gravel indentations in landfills. Eldesouky et al. [15] evaluated a GMB strain developed from bubbles beneath the GMB in pond liner applications using 3D nonlinear, finite-element analysis, and examined how GMB stiffness, fluid depth, volume of entrapped gas, and interface friction affect the deformed shape and maximum strain. Marcotte and Fleming [16] utilized close-range photogrammetry to measure vertical and lateral displacements of square grids drawn on the GMB, yet the accuracy relied on the grid size. The local strains induced by cushion layers depend on vertical stress, particle size and particle-size distribution of the cushion layer. Previous studies have investigated the strain calculation methods of HDPE GMBs in landfills and ponds on a specific cushion layer adopting a linear strain, without considering the effect of gradation parameters on the strains. This study investigates the strains of PVC-P GMBs induced by the granular cushions in pumped storage power stations, the areal strain was proposed to evaluate the strains on flat-rounded gravel gradations, the void width–depth ratio was adopted to correlate with strains, and the gradation limits were derived based on an allowable strain criterion. These aspects are genuinely new, and the findings would provide valuable references for practical engineering.

In this paper, a self-developed testing apparatus for GMB deformation and a 3D laser scanner were adopted to investigate the deformation behavior of PVC-P GMBs resting on porous medium cushions. A model correlating the areal strain of the GMB with void parameters and gradation indices was established, and the gradation parameters of flat-rounded gravel cushions satisfying the deformation requirements were proposed. The objectives are outlined as follows: (1) Conduct local deformation tests of PVC-P GMBs on uniform cushions under various pressure levels, and establish a mathematical model relating the linear strain and areal strain of PVC-P GMBs with void width–depth ratio to select an appropriate strain evaluation index. (2) Perform deformation tests on PVC-P GMBs laid on flat-rounded gravel cushions with varying gradations to explore the relationship between the areal strain and gradation parameters. (3) Identify the gradation parameters of flat-rounded gravel cushions satisfying the deformation requirements. The findings will provide insight into the deformation behavior of PVC-P GMBs on cushion layers, providing valuable evidence for cushion layer design and improving the safety and long-term durability of GMB lining systems for pumped storage power stations.

## 2. Materials and Methods

### 2.1. Materials

#### 2.1.1. PVC-P GMB

The experiment employed PVC-P GMBs produced by a manufacturer (Xingwei Geosynthetics Co., Ltd., Wuxi, China), with each roll measuring 50.0 m in length and 2.0 m in width. The physical and mechanical properties are presented in Table 1.

#### 2.1.2. Lead Sheet

For the tests, 0.5 mm thick high-purity lead sheets manufactured by a metal products company in Liaocheng, Shandong Province, were adopted to record the final deformed shape of PVC-P GMBs on different particle cushions. The physical and mechanical properties of lead sheet are summarized in Table 2.

#### 2.1.3. Uniform Granular Cushions

**Figure 1:**
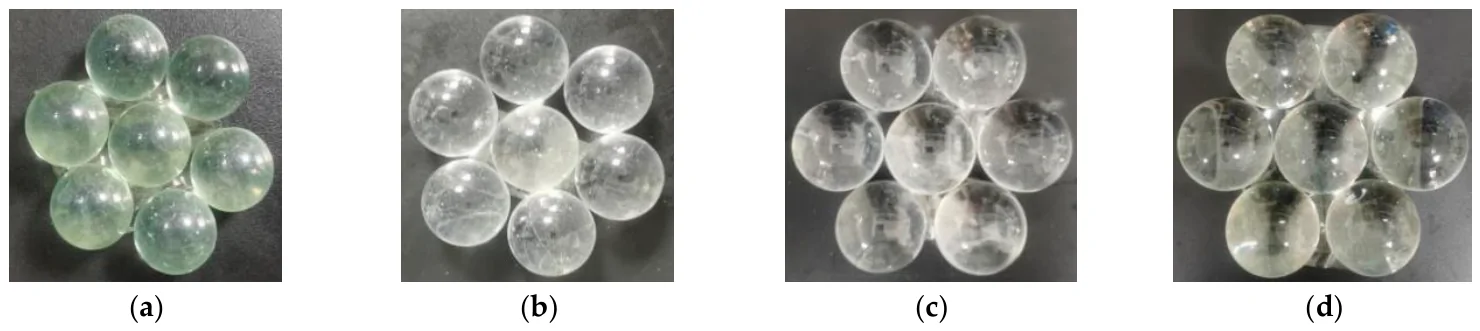
Glass spheres used in the test: (**a**) 20 mm; (**b**) 30 mm; (**c**) 40 mm; (**d**) 50 mm.

Transparent glass spheres with smooth surfaces and negligible impurities were selected as uniform granular cushions in this study. The spheres are primarily composed of silicon dioxide (SiO_2_), with diameters of 20, 30, 40, and 50 mm, as shown in Figure 1. The parameters of transparent glass spheres are listed in Table 3.

#### 2.1.4. Flat-Rounded Gravel Cushions

**Figure 2:**
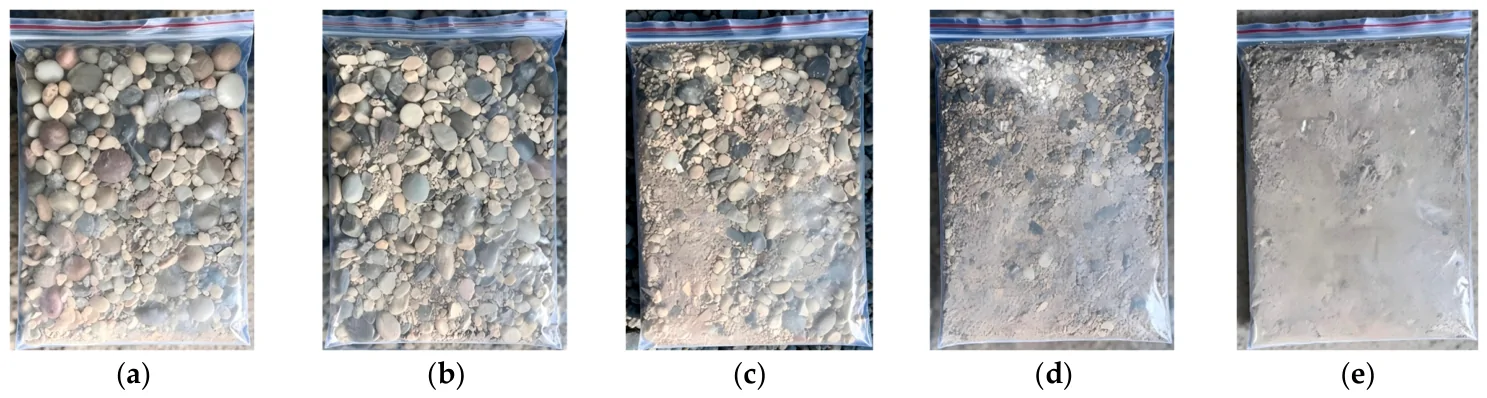
Schematic of flat-rounded gravel cushions: (**a**) JP1; (**b**) JP2; (**c**) JP3; (**d**) JP4; (**e**) JP5.

**Figure 3:**
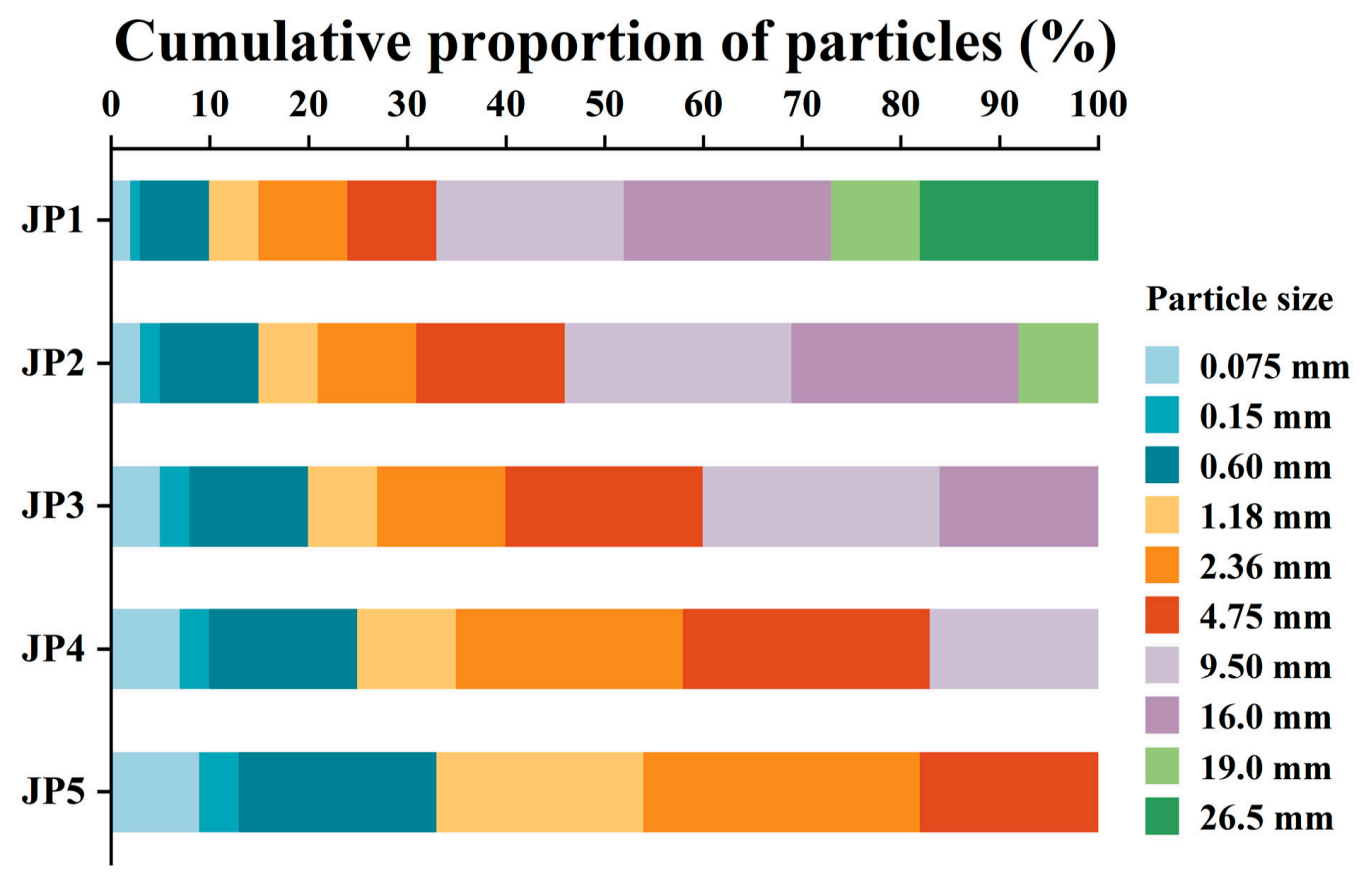
Cumulative proportion distribution of particle of flat-rounded gravel cushions.

The gradation parameters, such as maximum limiting particle size (*D*_max_), non-uniformity coefficient (*C*_u_) and coefficient of curvature (*C*_c_) are reported in Table 4. The schematic and cumulative particle proportion of the flat-rounded gravel cushions are provided in Figure 2 and Figure 3, respectively.

### 2.2. Test Apparatus

#### 2.2.1. Test Apparatus for GMB Deformation over Porous Medium Cushions

**Figure 4:**
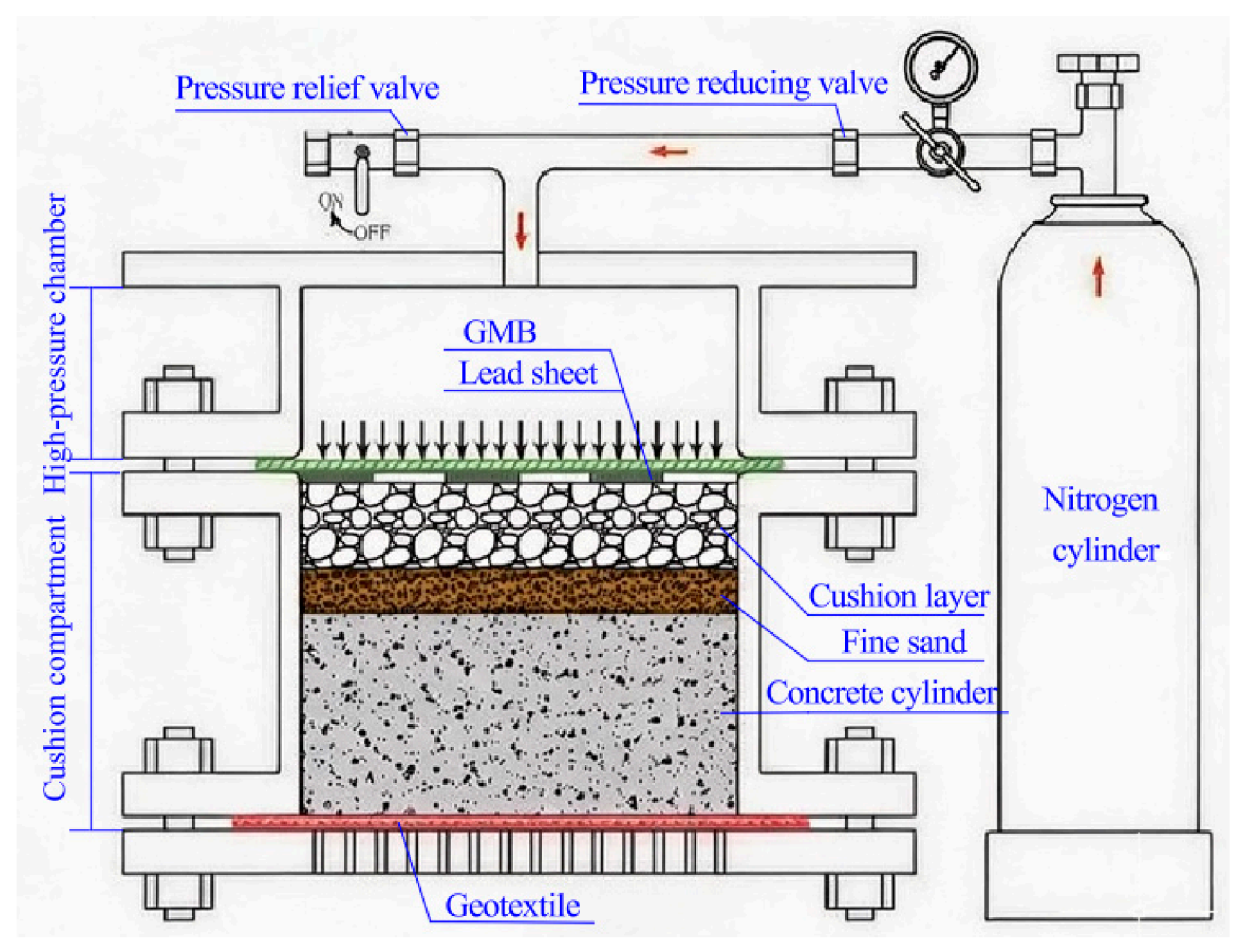
Deformation tester for GMB on porous media cushion.

This study employs a self-developed test apparatus for GMB deformation over porous medium cushions. The apparatus adopts a cylindrical pressure vessel with an inner diameter of 200 mm and mainly consists of a high-pressure chamber and a cushion compartment, as illustrated in Figure 4. The left side of the high-pressure chamber top is connected to a pressure relief valve, and the right side is connected to a pressure control system. The pressure relief valve releases the gas pressure inside the high-pressure chamber after each test. The pressure control system is composed of a nitrogen cylinder and a pressure reducing valve, and the pressure reducing valve regulates the gas pressure delivered from the nitrogen cylinder into the high-pressure chamber. From top to bottom, the cushion compartment is arranged with a 1.5 mm thick PVC-P GMB, a 0.5 mm thick lead sheet, a granular cushion with a thickness of 10–50 mm, 10–50 mm thick fine sand, a plain concrete cylinder (170 mm in height and 200 mm in diameter), and a 600 g/m^2^ nonwoven needle-punched geotextile. The plain concrete cylinder is adopted to simulate rigid components in the GMB lining system of pumped storage reservoir basins, preventing the cylinder from settlement under pressures ranging from 0.2 to 0.8 MPa. The geotextile acts as a buffer layer to maintain the integrity of the plain concrete cylinder.

#### 2.2.2. Handheld 3D Laser Scanner

Following the deformation test, the deformed lead sheet was scanned using a handheld 3D laser scanner (SIMSCAN22, manufactured by Sikan Technology Co., Ltd., Hangzhou, China) to capture surface topography for quantitative geometric analysis. The technical parameters of the handheld 3D laser scanner are provided in Table 5.

### 2.3. Test Methods

#### 2.3.1. Test Scheme

As a primary anti-seepage barrier in the upper reservoir of pumped storage power stations worldwide, PVC-P GMBs routinely experience hydraulic heads of 20 to 80 m. Consistent with this service condition, the deformation response of PVC-P GMBs on cushion layers was investigated across four pressure levels: 0.2, 0.4, 0.6, and 0.8 MPa.

To investigate the effect of granular cushions on the strain of PVC-P GMBs, nine cushions were adopted in the tests, including four groups of uniform cushions with particle diameters of 20, 30, 40, and 50 mm, and five groups of graded flat-rounded gravel cushions. Tests under four different test pressures were carried out for each cushion, yielding a total of 36 test cases. Three replicate tests were performed for each test condition, and the recorded values were averaged as the final result.

#### 2.3.2. Test Procedure

**Figure 5:**
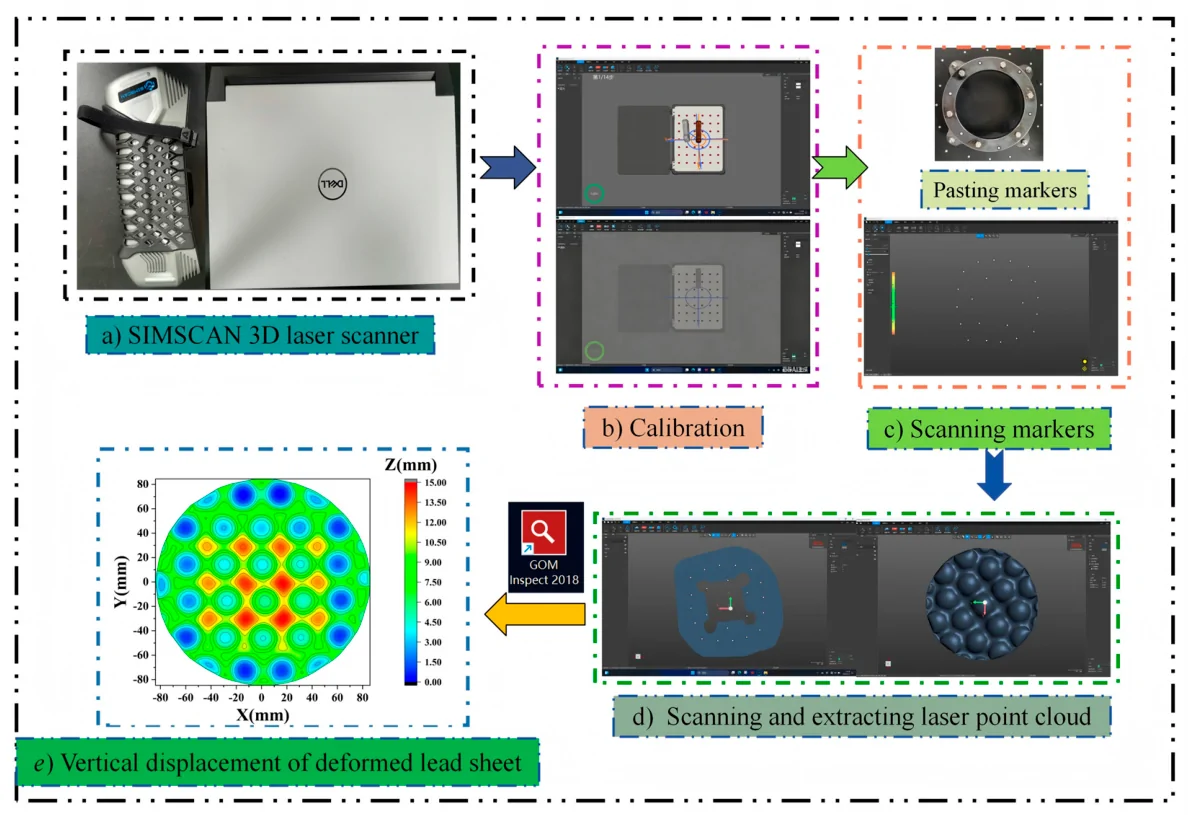
Flow chart of scanning and data analysis.

The tests were implemented in two stages. First, a testing apparatus for GMB deformation on a porous medium cushion was employed to conduct deformation tests on PVC-P GMBs under various pressures and cushions, and lead sheets recording the deformation of PVC-P GMBs were obtained. Second, a SIMSCAN handheld 3D laser scanner was utilized to quantify the deformation of lead sheets and acquire 3D point cloud data. Subsequently, data analysis was performed via GOM Inspect 2018 software to calculate the strain of PVC-P GMBs. The flow chart of scanning and data analysis is plotted in Figure 5. The detailed procedures are described below.

##### PVC-P GMB Deformation Tests

(1) Specimens with diameters of 280 mm were cut within the region 100 mm from the edge of the PVC-P GMB roll. The specimens were conditioned for 24 h at a temperature of 20 ± 2 °C and relative humidity of 60 ± 10% prior to testing.
(2) A needle-punched nonwoven geotextile with a diameter of 280 mm and mass per unit area of 600 g/m^2^ was placed on the porous flange plate, which was connected to the cushion compartment using bolts. A concrete cylinder with a diameter of 200 mm was placed inside the cushion compartment, followed by fine sand, which was compacted using a cylindrical rammer with a diameter of 50 mm. Afterwards, the granular cushion, lead sheet and PVC-P GMB specimen were laid sequentially. The cushion compartment and high-pressure chamber were fastened with bolts to clamp the PVC-P GMB specimen.
(3) The pressure relief valve was closed, and the nitrogen cylinder valve and pressure reducing valve were opened to apply the pressure. Once the desired pressure was reached, it was held for 24 h.
(4) Upon completion of the test, the nitrogen cylinder valve and pressure reducing valve were closed, and the pressure relief valve was opened to vent gas inside the high-pressure chamber until atmospheric pressure was reached. The deformed lead sheet was retrieved and placed into transparent sealed bags for subsequent scanning.

##### Scanning Deformed Lead Sheets and Data Analysis

(1) The horizontal holder was leveled by adjusting the base nuts and calibrating with a spirit level. Subsequently, the lead sheet was retrieved from the transparent sealed bag and clamped on the horizontal holder with bolts.
(2) The handheld 3D laser scanner captured the reference plate from 14 positions in sequence following the on-screen instructions to implement calibration, and the measurement accuracy was automatically calibrated by the software.
(3) Markers of 6 mm diameter were uniformly pasted within a 5 cm perimeter surrounding the horizontal holder. The marker scanning mode was activated in ScanViewer. Subsequently, the handheld 3D laser scanner identified the markers from multiple orientations. (e.g., directly above, 45° forward/backward/leftward/rightward) until all markers turned white on the interface.
(4) Laser sheet mode was selected to scan the lead sheet from multiple angles using the handheld 3D laser scanner. The scanned results were saved in the *.pj format. The laser point cloud data was extracted adopting the tools including “Connection” and “Lasso” in ScanViewer. Finally, the data was encapsulated into mesh files in .stl format.
(5) The laser point cloud mesh files were imported into GOM Inspect 2018 software. The area and lengths of the lead sheets before and after deformation were obtained using tools such as “Construct” and “Inspect”. Subsequently, the linear strain and areal strain of PVC-P GMBs were calculated and imported into Origin 2018 to investigate the deformation behavior of PVC-P GMBs on porous medium cushions.

### 2.4. Analytical Methods

#### 2.4.1. Principle of Equivalent Circle

**Figure 6:**
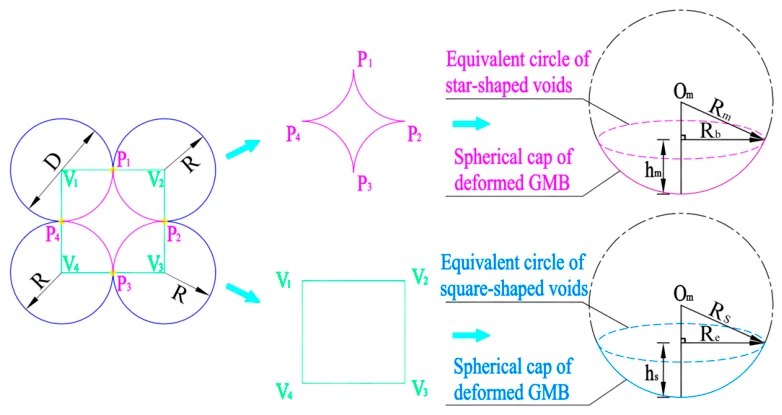
Principle of void equivalent circle.

This study introduces the equivalent circle principle for voids. Specifically, the star-shaped void formed by four particles in the cushion layer is converted into an equivalent circle with identical area, and the deformation of the GMB on cushion layers is assumed to form a spherical crown surface. The star-shaped plane defined by points P_1_ to P_4_ is regarded as the initial deformation plane of the PVC-P GMB. Nevertheless, in practical engineering, the initial deformation plane of the PVC-P GMB over particle cushions is the square plane formed by vertices V_1_ to V_4_ of the four particles. Accordingly, the equivalent circle principle for square-shaped voids is developed on the basis of the equivalent circle principle for star-shaped voids. Taking the square plane formed by V_1_ to V_4_ as the initial deformation plane, the square void is transformed into an equivalent circle of equal area, and the PVC-P GMB deforms into a spherical crown configuration. The principle of void equivalent circle is shown in Figure 6.

For square void, equivalent circle can be expressed as:

(1) $$R_{s}^{2}=R_{e}^{2}+{\left(R_{s}-h_{s}\right)}^{2}$$

(2) $$D^{2}=\pi {R_{e}}^{2}$$

where $R_{s}$ is the radius of the bulged sphere of the GMB, $h_{s}$ is the bulging height of the GMB, $R_{e}$ is the radius of the equivalent circle, D is the diameter of the granular cushion.

$R_{e}$ and $R_{s}$ are given by:

(3) $$R_{e}=\sqrt{\frac{A_{c}}{\pi }}$$

(4) $$R_{s}=\frac{A_{s}}{2\pi h_{s}}$$

where $A_{c}$ is the undeformed area of the GMB, $A_{s}$ is the deformed area of the GMB.

Combining Equations (1), (3) and (4) yields:

(5) $$h_{s}=\sqrt{\frac{A_{s}-A_{c}}{\pi }}$$

Thus, the width–depth ratio of the void $m_{s}$ can be expressed as:

(6) $$m_{s}=\frac{R_{s}}{h_{s}}$$

#### 2.4.2. Strain Calculation

The linear strain and areal strain of the GMB can be calculated as follows:

(7) $$\varepsilon_{L}=\frac{L_{1}-L_{0}}{L_{0}}$$

(8) $$\varepsilon_{A}=\frac{A_{1}-A_{0}}{A_{0}}$$

where $\varepsilon_{L}$ is the linear strain of the GMB, $L_{0}$ is the initial length passing through the center of the GMB, $L_{1}$ is the length passing through the apex of the bulging surface, $\varepsilon_{A}$ is the areal strain of the GMB, $A_{0}$ is the initial area of the GMB, $A_{1}$ is the deformed area of the bulging surface of the GMB.

#### 2.4.3. Analytical Process

For uniform granular cushions, the width–depth ratio was calculated based on the equivalent circle principle. The linear strain and areal strain were calculated based on the actual deformation data from 3D scanning. The relationship between linear strain and areal strain and width–depth ratio was then acquired.

For flat-rounded gravels, the width–depth ratio was derived from actual measurements, and the areal strain was computed from scanner-acquired data. The relationship was identified between areal strain and width–depth ratio. Given that the width–depth ratio was correlated with cushion gradation parameters, the width–depth ratio and corresponding cushion gradation were then determined for a specified strain criterion.

## 3. Results and Discussion

### 3.1. Deformation Tests of PVC-P GMBs on Uniform Granular Cushions

#### 3.1.1. Full-Field Vertical Displacement

The 3D point cloud data of the deformed lead sheet were extracted using ScanViewer 6.3.3.13 software bundled with the handheld 3D laser scanner. The origin of the coordinate system was defined at the center of the interface between the undeformed lead sheet and the uniform cushion layer. It is stipulated that the vertical displacement in the downward direction of the plane is positive.

**Figure 7:**
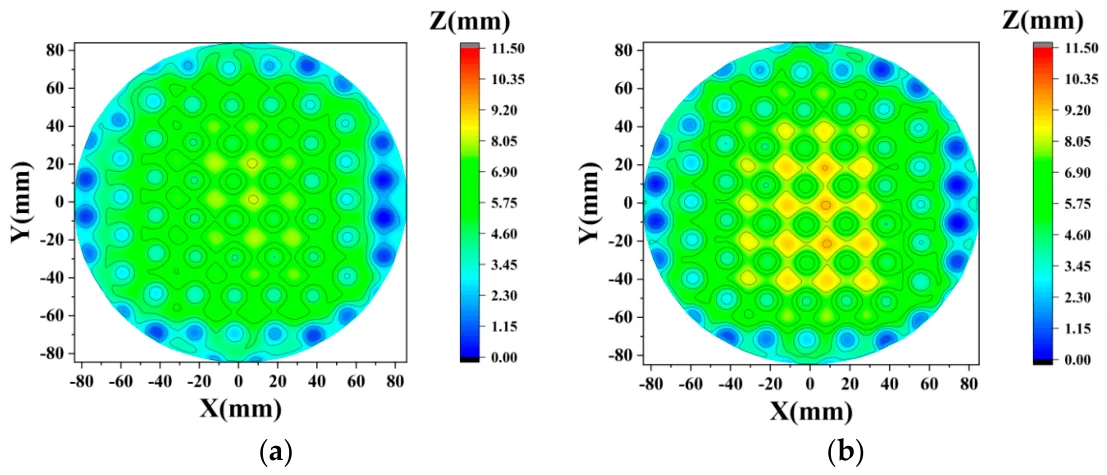

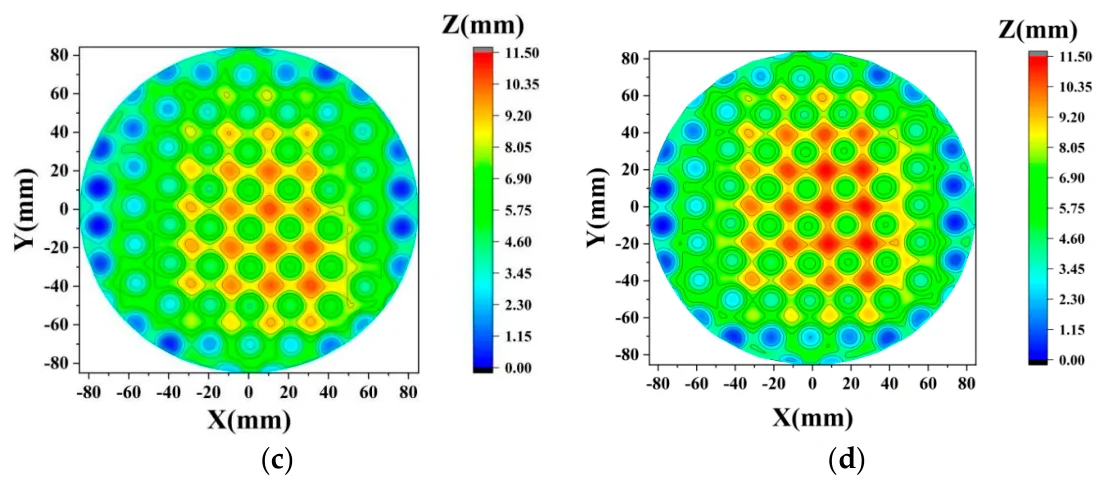
Vertical displacement contours of the PVC-P GMB on the 20 mm glass spheres: (**a**) 0.2 MPa; (**b**) 0.4 MPa; (**c**) 0.6 MPa; (**d**) 0.8 MPa.

**Figure 8:**
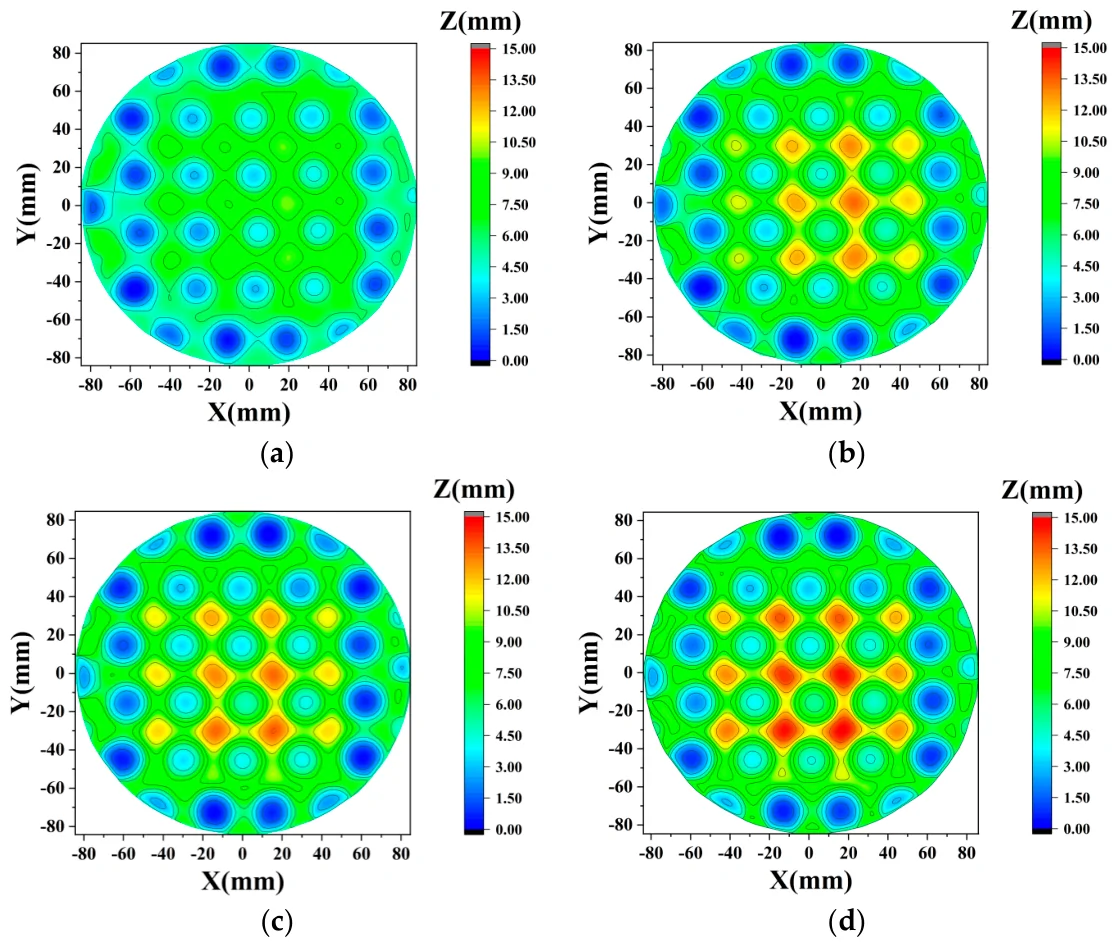
Vertical displacement contours of the PVC-P GMB on the 30 mm glass spheres: (**a**) 0.2 MPa; (**b**) 0.4 MPa; (**c**) 0.6 MPa; (**d**) 0.8 MPa.

**Figure 9:**
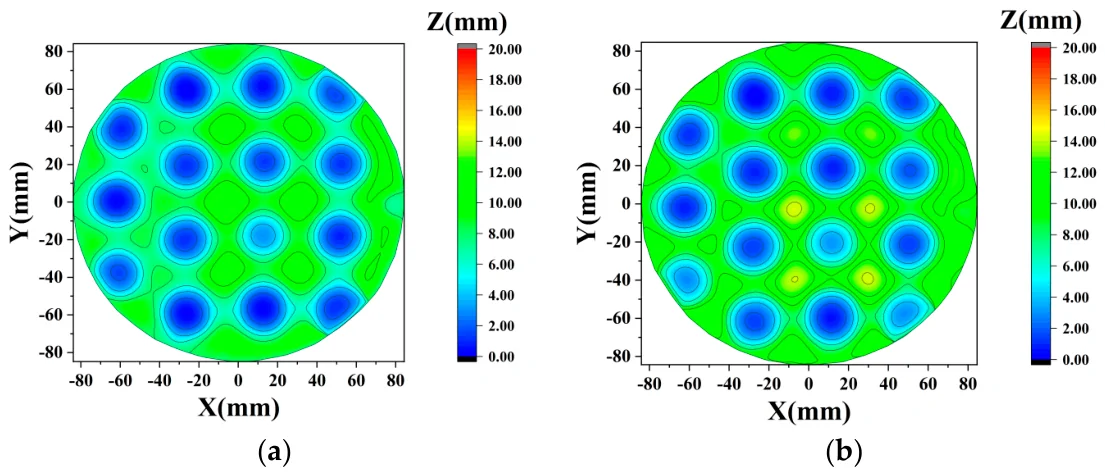

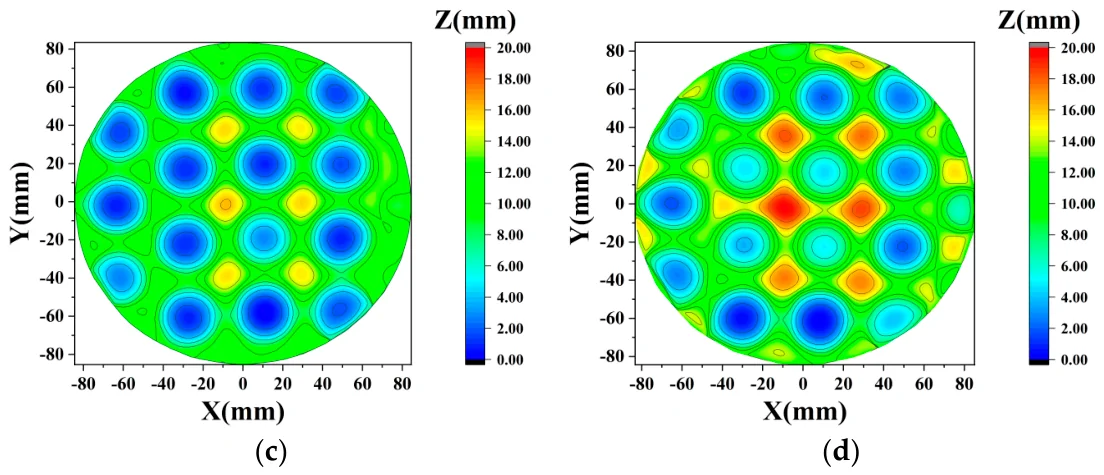
Vertical displacement contours of the PVC-P GMB on the 40 mm glass spheres: (**a**) 0.2 MPa; (**b**) 0.4 MPa; (**c**) 0.6 MPa; (**d**) 0.8 MPa.

**Figure 10:**
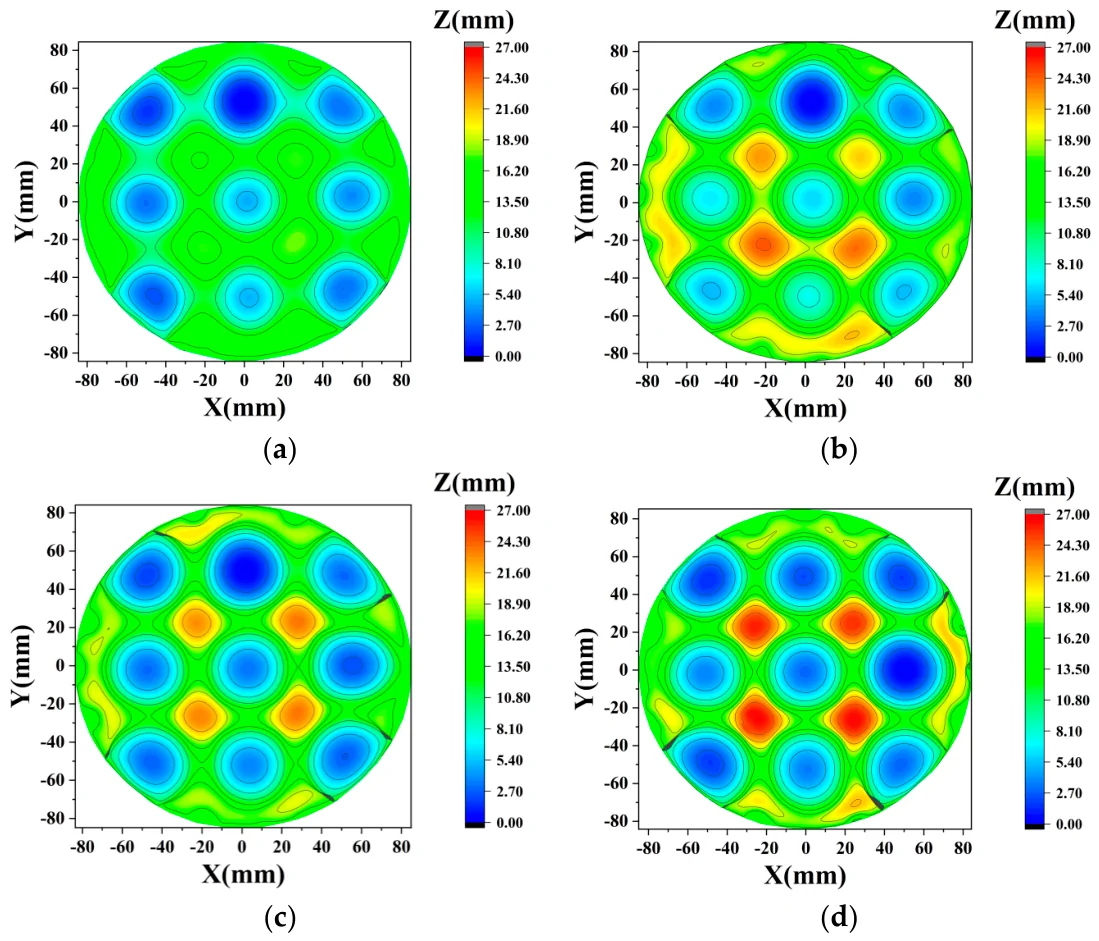
Vertical displacement contours of the PVC-P GMB on the 50 mm glass spheres: (**a**) 0.2 MPa; (**b**) 0.4 MPa; (**c**) 0.6 MPa; (**d**) 0.8 MPa.

Figure 7, Figure 8, Figure 9 and Figure 10 report the vertical displacement contours of PVC-P GMBs on varying cushion layers at tested pressures. The distribution trends of vertical displacement with pressure across the four particle sizes were largely consistent. Larger vertical displacements were observed within inter-particle voids, whereas smaller displacements were concentrated at the interface between the PVC-P GMB and the cushion layer. For a specific diameter, vertical displacements increased progressively with applied pressure. For instance, the maximum vertical displacement for 40 mm glass spheres increased from 11.99 mm at 0.2 MPa to 19.76 mm at 0.8 MPa. Under identical pressures, vertical displacements increased with increasing particle diameter. At 0.6 MPa, the peak vertical displacements reached 10.63, 13.55, 16.23, and 23.68 mm for 20, 30, 40, and 50 mm cushions, respectively. This phenomenon is attributed to deeper inter-particle voids created by coarser particles, providing greater space for local deformation within the PVC-P GMB.

Owing to boundary constraints, the deformation on the edge of lead sheet was suppressed, leading to minimal vertical displacements. Additionally, the displacement field exhibited pronounced heterogeneity, and large deformations were concentrated in the central zone. For engineering safety considerations, the point cloud data within a central 40 mm × 40 mm square region were adopted to calculate the local linear strain, local areal strain, and void width–depth ratio.

#### 3.1.2. Vertical Displacement on the Characteristic Cross-Section

**Figure 11:**
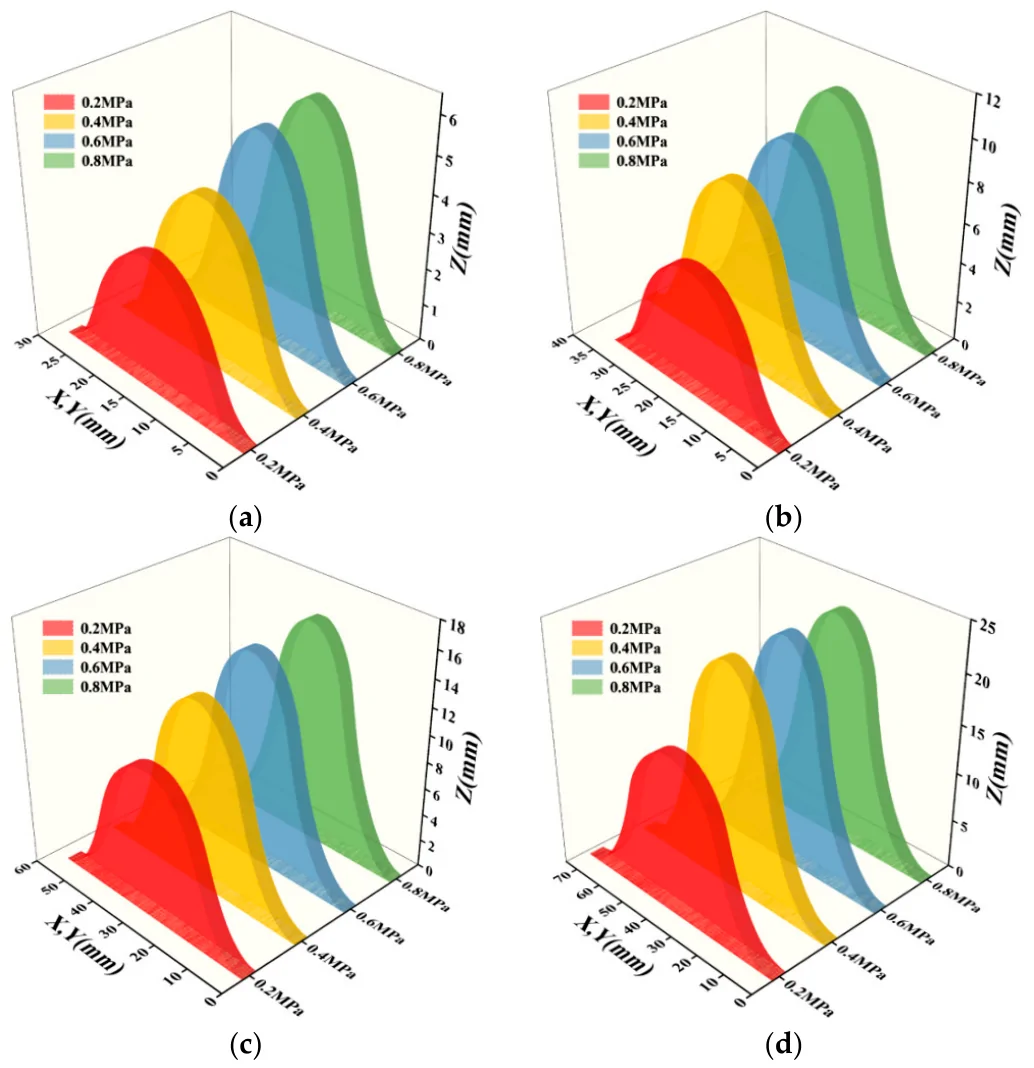
Vertical displacement of deformed PVC-P GMB on the diagonal of the voids on the uniform glass spheres: (**a**) 20 mm; (**b**) 30 mm; (**c**) 40 mm; (**d**) 50 mm.

3D coordinates along the diagonals of square voids formed by four particles were extracted to generate 3D wall-shaped plots. Figure 11 presents the vertical displacement profiles of the PVC-P GMB along the diagonal of voids for uniform glass spheres of different diameters. For a given particle size, the vertical displacement increased continuously with the applied pressure. Under identical pressures, larger particle sizes produced wider inter-particle voids and induced higher vertical displacements. The maximum deformation occurred at the center of void diagonals, while displacement decayed rapidly towards particle supporting points. These results demonstrate that excessively large particle sizes aggravate local GMB deformation and increase the tensile failure risk.

#### 3.1.3. Local Strain

**Figure 12:**
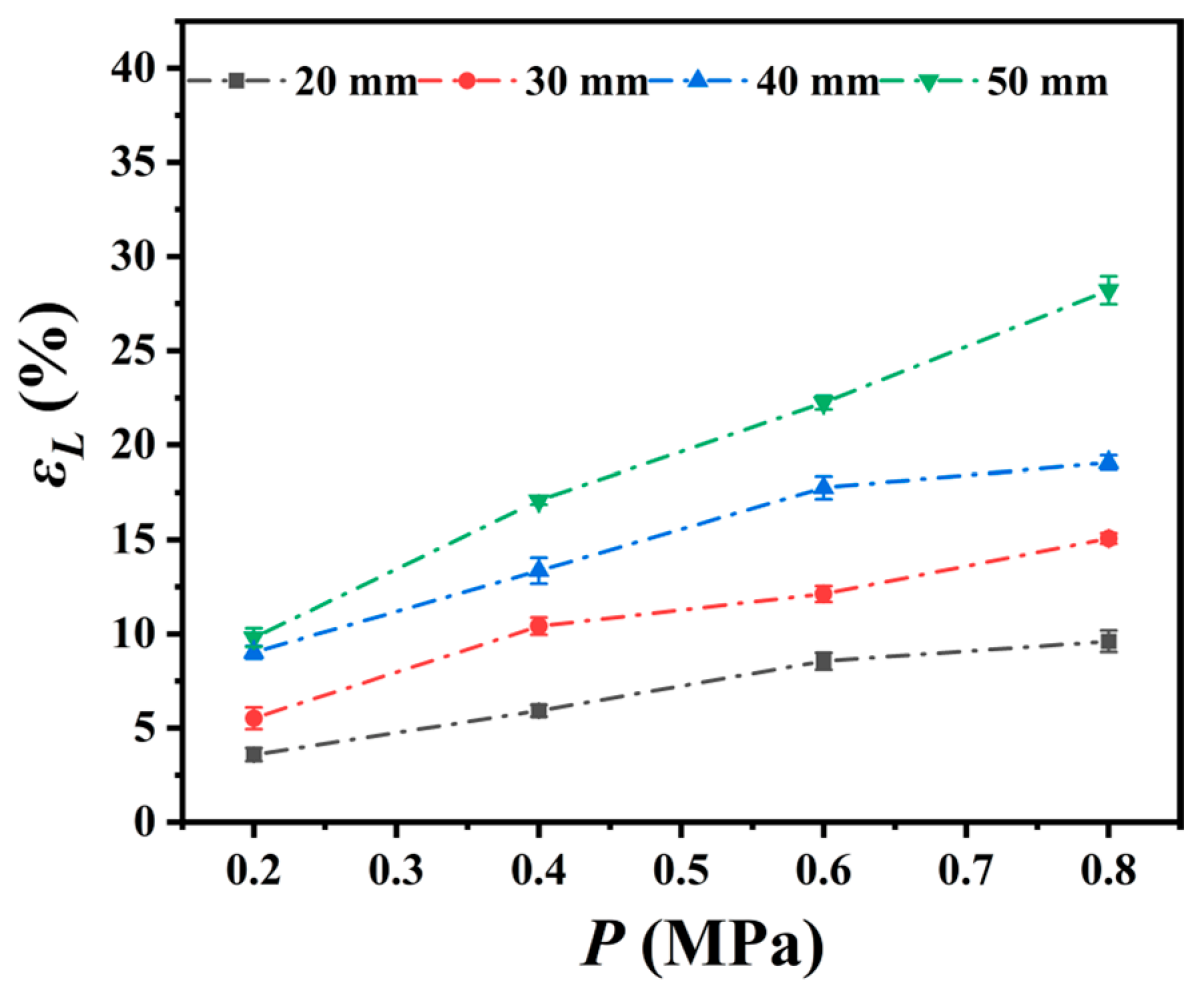
Relationship between local linear strain and applied pressure for uniform cushions of varying diameters.

**Figure 13:**
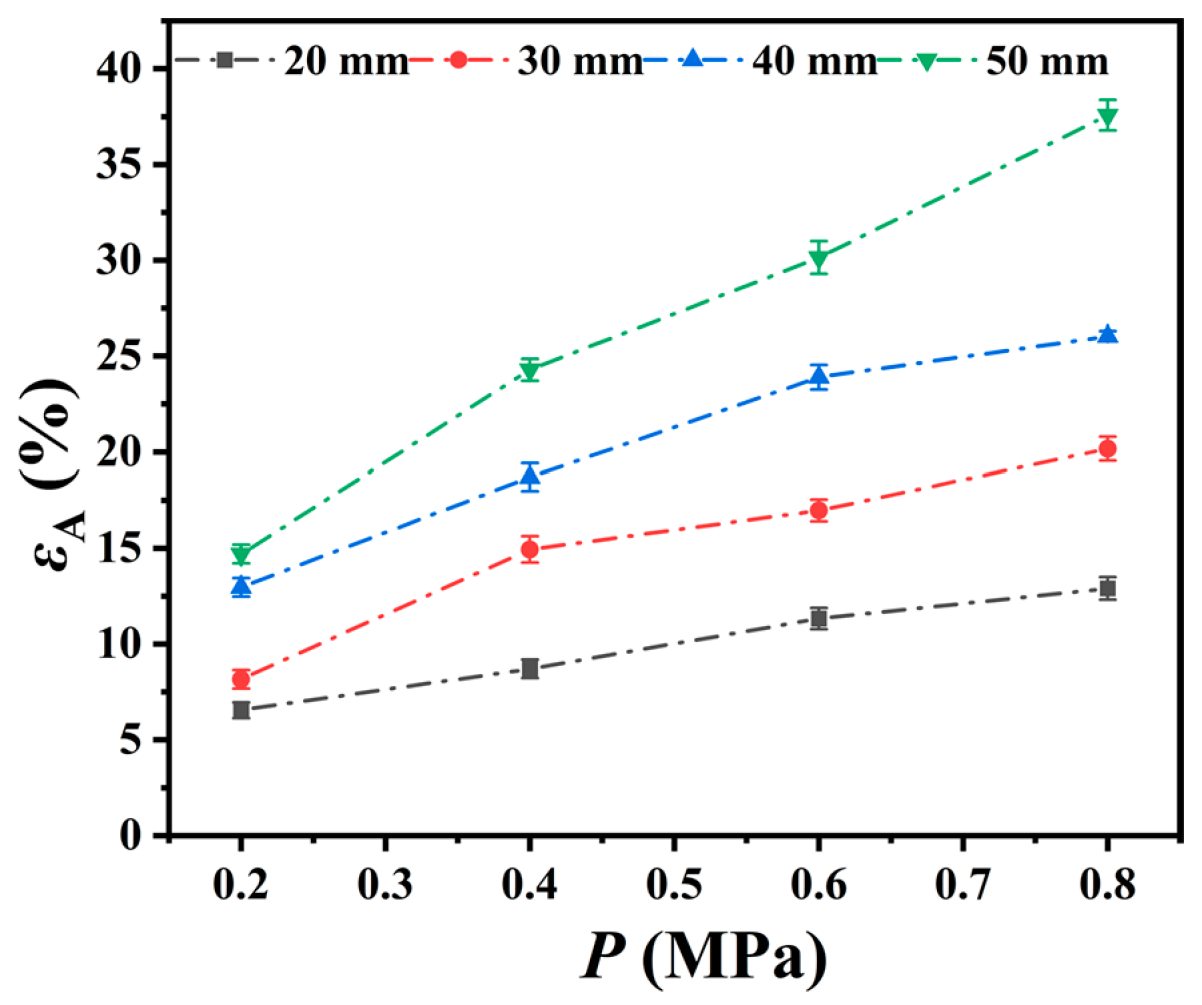
Relationship between local areal strain and applied pressure for uniform cushions of varying diameters.

Local linear strain and local areal strain were calculated using Equations (7) and (8). Figure 12 reports the variation in local linear strain of PVC-P GMBs with pressure for cushions of varying diameters. For all tested cushions, the local linear strain increased monotonically with the increase in pressure. For the 30 mm cushion layer, the linear strain increased from 5.52% (0.2 MPa) to 15.06% (0.8 MPa). Under identical pressure levels, larger particle diameters led to higher local linear strains. At 0.8 MPa, the linear strains of cushions with particle diameters of 20, 30, 40, and 50 mm were 9.60%, 15.06%, 19.07%, and 28.21%, respectively. The linear strain under the 50 mm cushion layer was 2.94 times that under the 20 mm condition. This is because the voids formed between coarse particles provide considerable deformation space for the GMB. Figure 13 depicts the relationship between local areal strain of PVC-P GMBs and pressure for cushions of varying diameters. The local areal strain followed similar patterns to the local linear strain, exhibiting an increasing trend with increasing particle size and pressure. The observed increase in local strain with increasing particle size and pressure in this study was consistent with the findings reported by Cen et al. [20] that both the increasing particle size and the higher applied pressure contributed to larger vertical displacements and tensile strains of HDPE GMBs. However, the local areal strain was higher than the local linear strain under identical conditions. For the 30 mm cushion layer at 0.6 MPa, the local areal strain of 18.69% was 1.40 times that of the corresponding local linear strain of 13.35%, consistent with the relationship between areal strain and linear strain reported by Shu [21] that the areal strain was approximately 2 times that of the linear strain.

**Figure 14:**
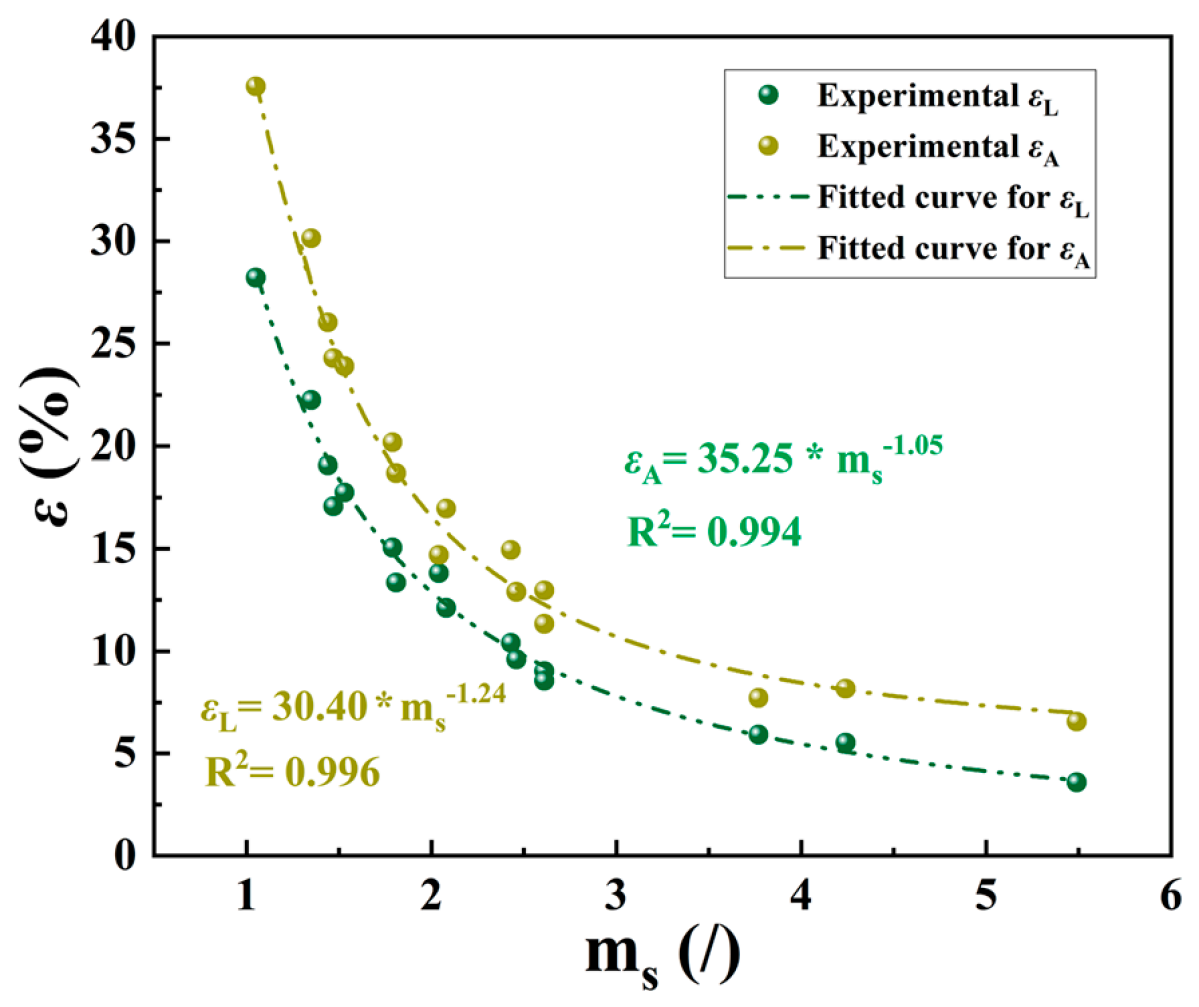
Relationships between local linear strain and local areal strain and void width–depth ratio for PVC-P GMBs on uniform spherical cushions.

The local linear strain and local areal strain of PVC-P GMB supported by uniform spherical cushions were fitted to the void width–depth ratio using the allometric function, as illustrated in Figure 14. The corresponding fitted equations were expressed as:

(9) $$\varepsilon_{L}=30.40*m_{s}^{-1.24}$$

(10) $$\varepsilon_{A}=35.25*m_{s}^{-1.05}$$

Regression analyses were performed for local linear and areal strain with 16 data points each. All fitted parameters were statistically significant (*p* < 0.05), and the determination coefficients were all greater than 0.991. The residual sum of squares (RSS) reached 0.2 for linear strain regression and 0.7 for areal strain regression, indicating satisfactory fitting performance and verifying the dependence of void width–depth ratio on the deformation behavior of PVC-P GMBs on granular cushions.

As observed, both the local linear strain and local areal strain decreased with increasing void width–depth ratio. Narrow and deep voids with small width–depth ratios induced remarkably higher strains in the GMB. For instance, the maximum areal strain was 37.57% at a void width–depth ratio of 1.05, while the minimum local areal strain equaled 6.55% at a width–depth ratio of 5.49. For a given width–depth ratio, the local areal strain was consistently larger than the local linear strain. The linear strain and areal strain decreased as the width–depth ratio increased, and the areal strain exceeded the linear strain, in agreement with the findings reported by Shu [21]. Since areal strain accounts for biaxial tensile effects and yields larger values compared with uni-axial linear strain, it is therefore recommended to evaluate the puncture risk of GMBs over particle cushions in practical engineering. However, the acquired linear strain and areal strain for the uniform cushions were based on the assumption of equivalent circle approach that the deformation can be represented by a spherical crown, which is a limitation of the analytical method. The areal strain follows an allometric function of the width–depth ratio. This relationship provides an analytical insight into evaluating the strain behavior of GMBs on flat-rounded gravel cushions. Therefore, the relationship between areal strain and width–depth ratio was investigated for PVC-P GMBs on flat-rounded gravel cushions.

### 3.2. Deformation Test of PVC-P GMB on Flat-Rounded Gravel Cushion

#### 3.2.1. Vertical Displacement

**Figure 15:**
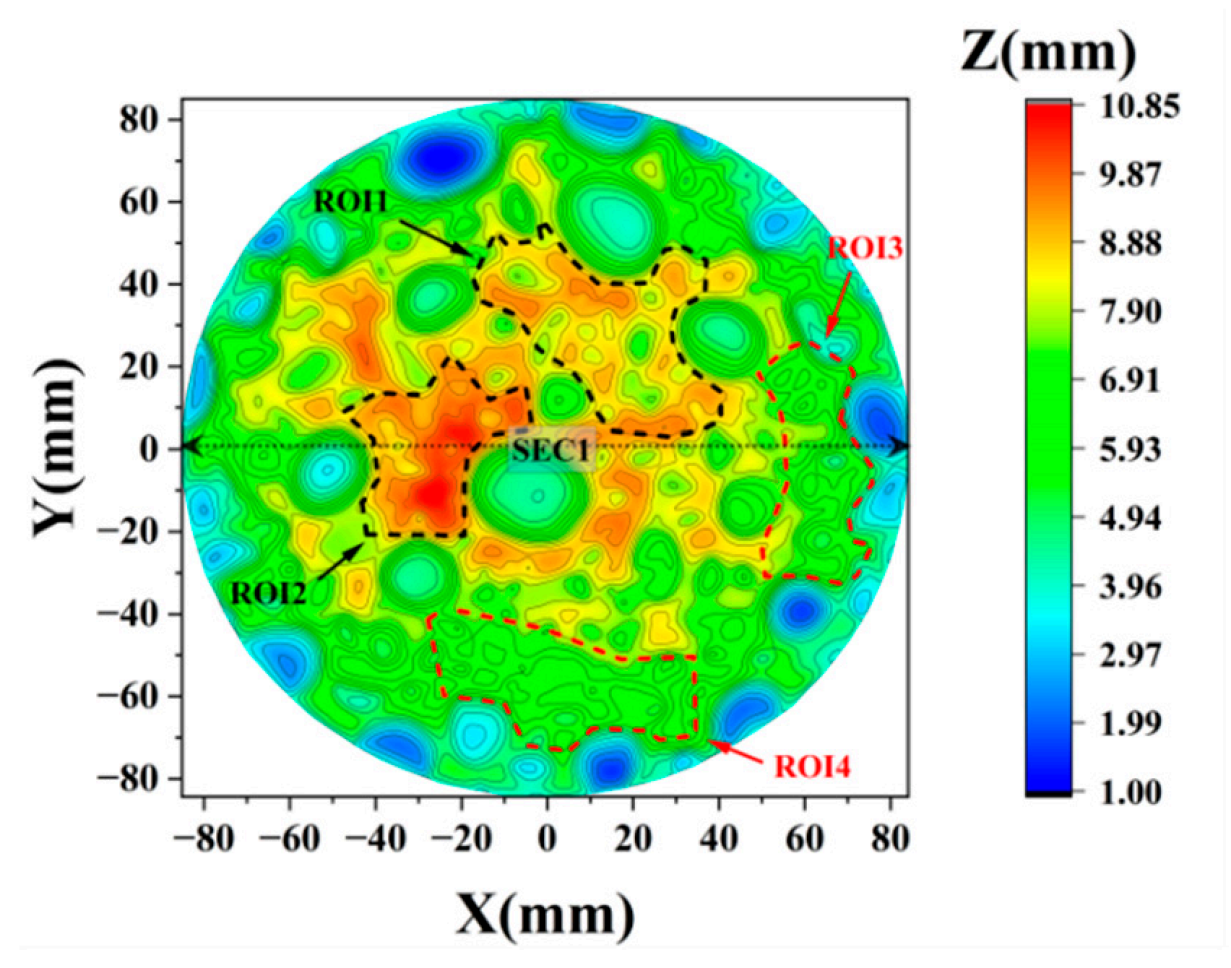
Vertical displacement contours of PVC-P GMB on JP1 cushion layer.

**Figure 16:**
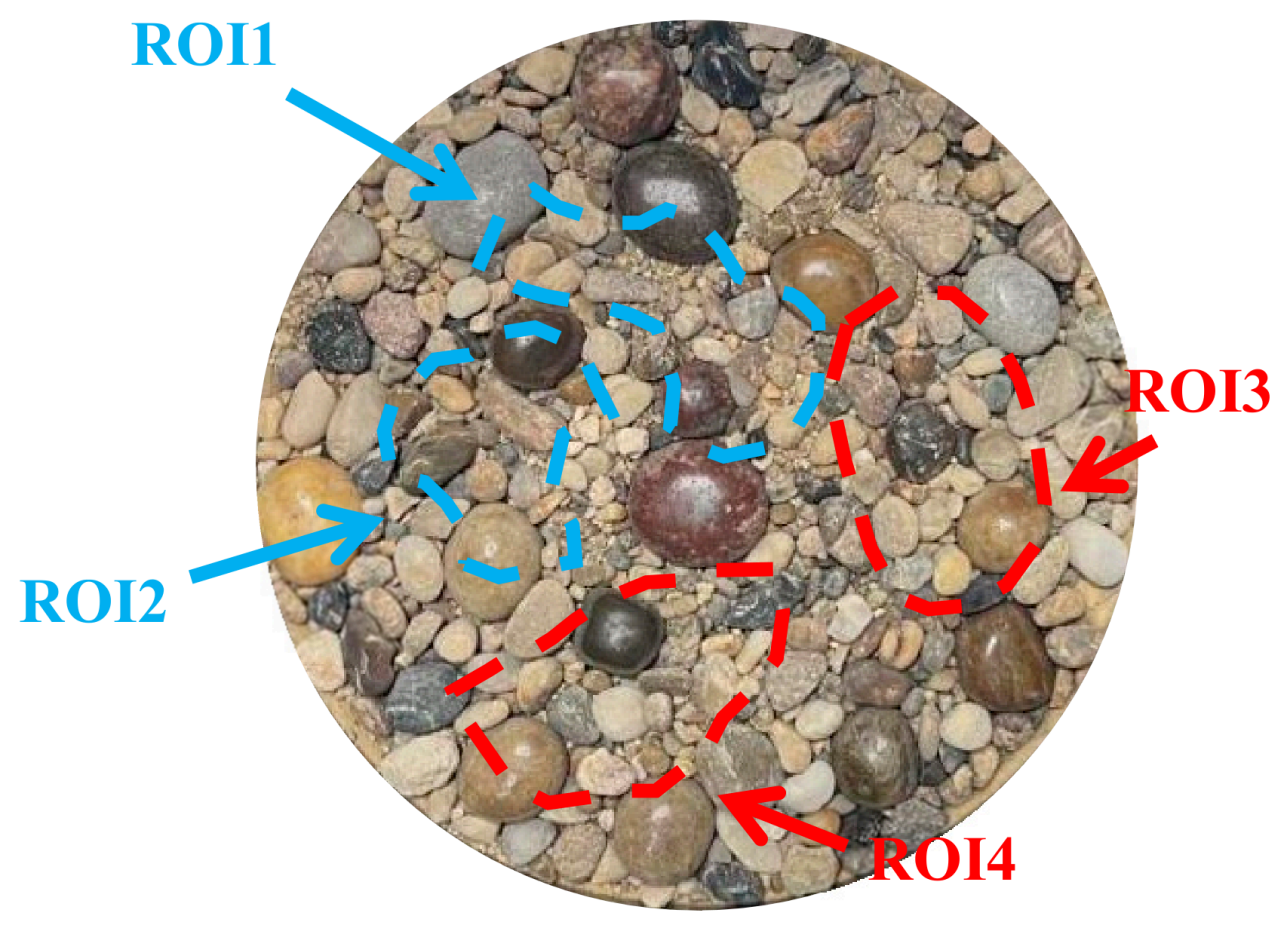
Layout of JP1 cushion layer.

**Figure 17:**
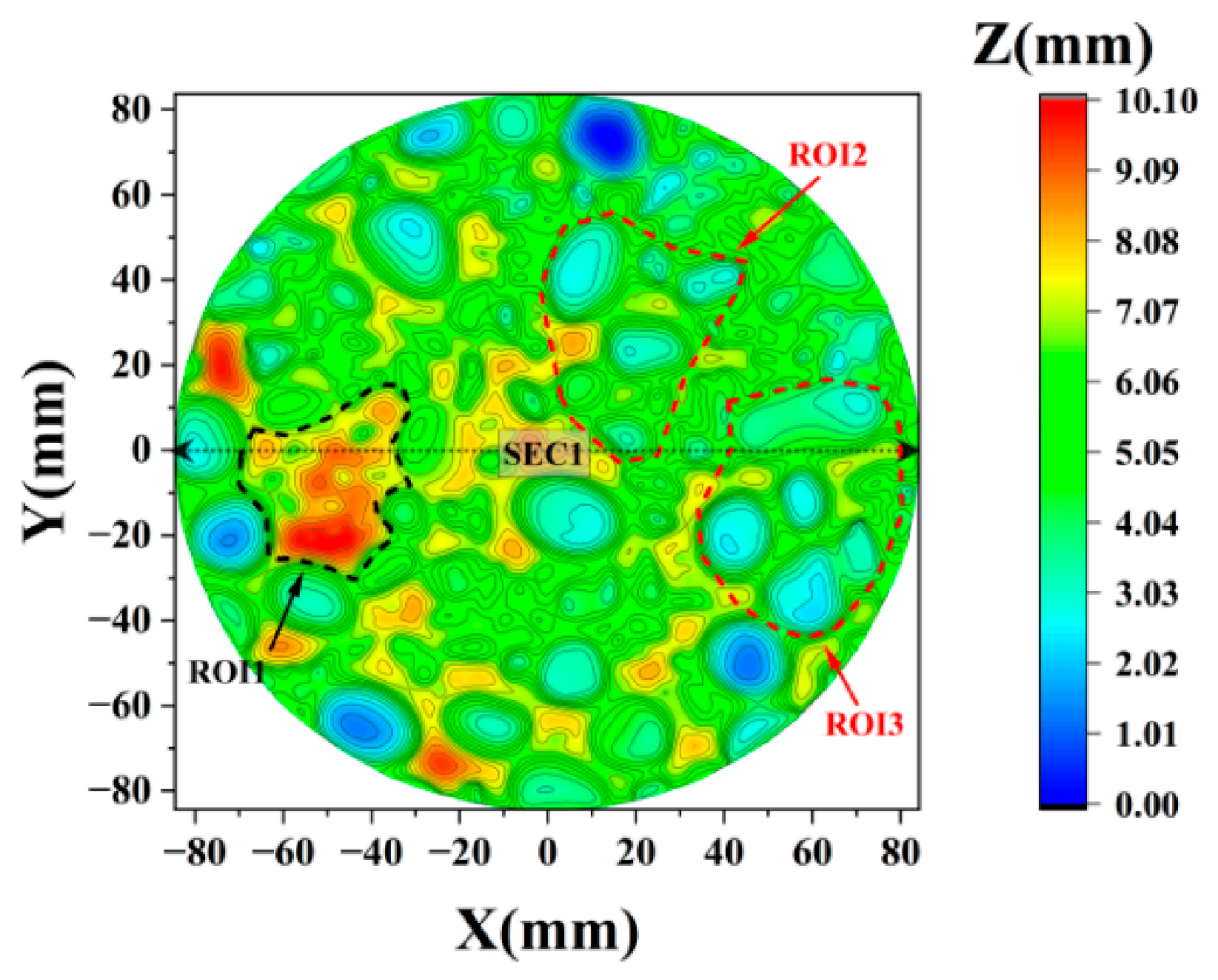
Vertical displacement contours of PVC-P GMB on JP2 cushion layer.

**Figure 18:**
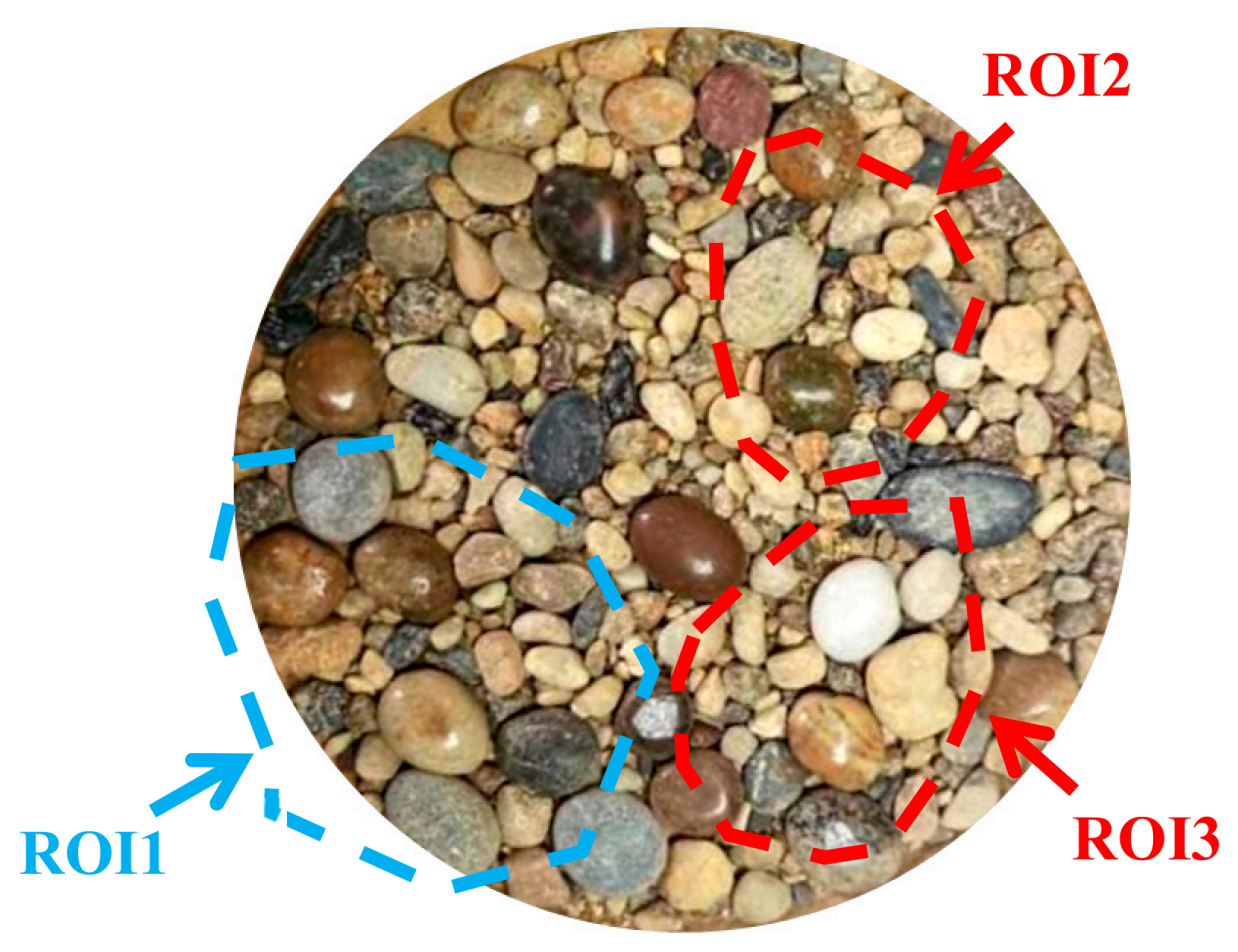
Layout of JP2 cushion layer.

**Figure 19:**
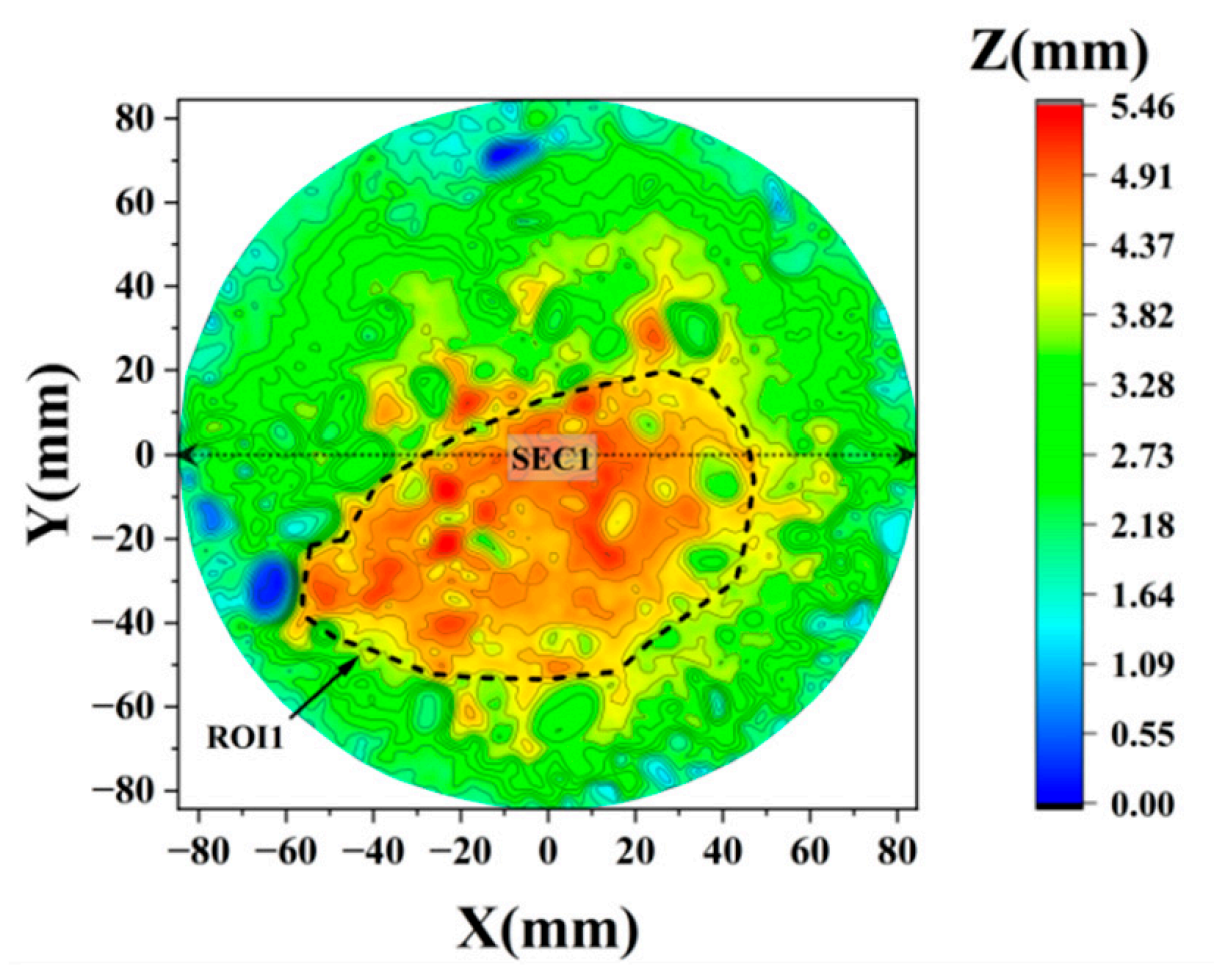
Vertical displacement contours of PVC-P GMB on JP3 cushion layer.

**Figure 20:**
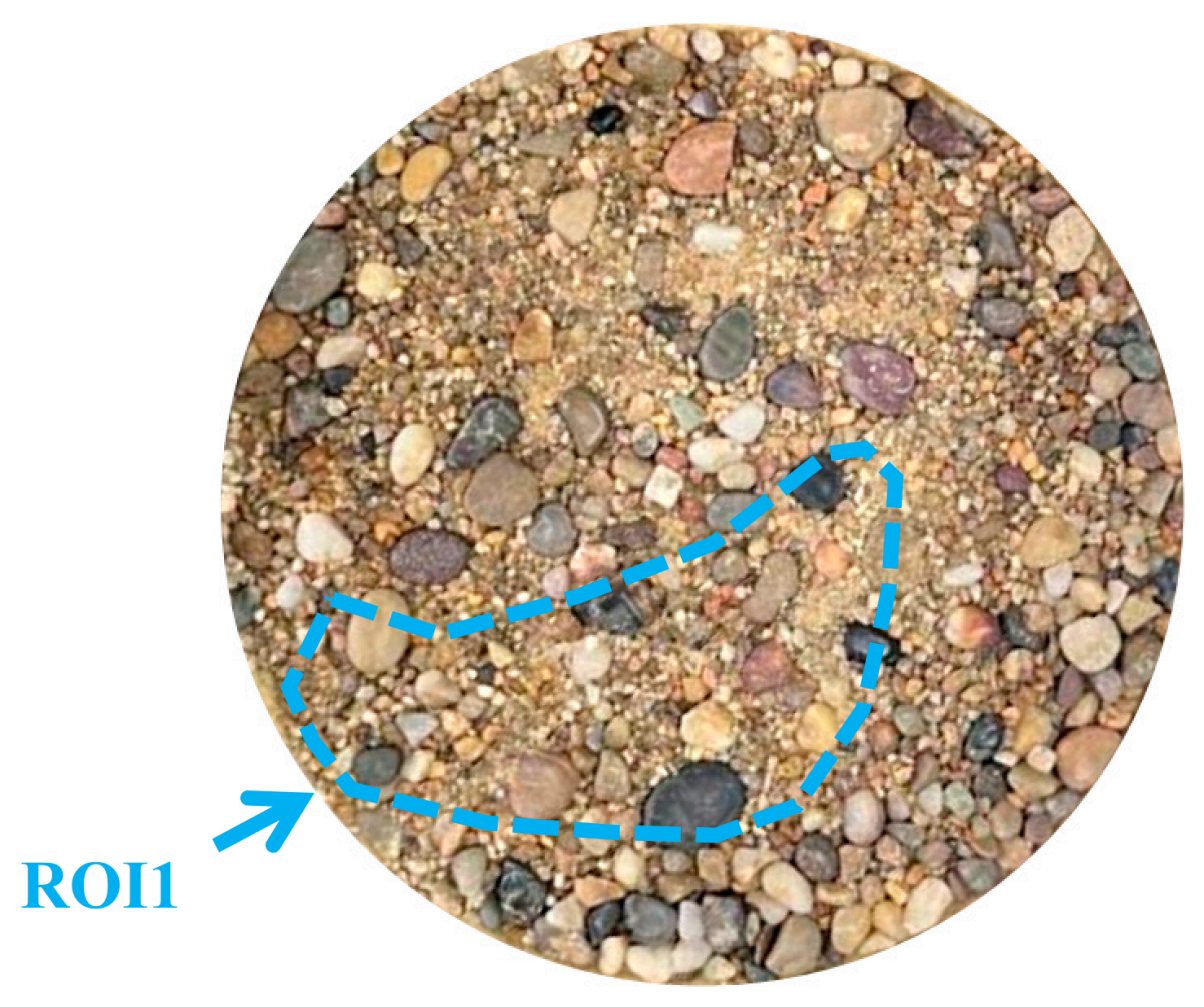
Layout of JP3 cushion layer.

**Figure 21:**
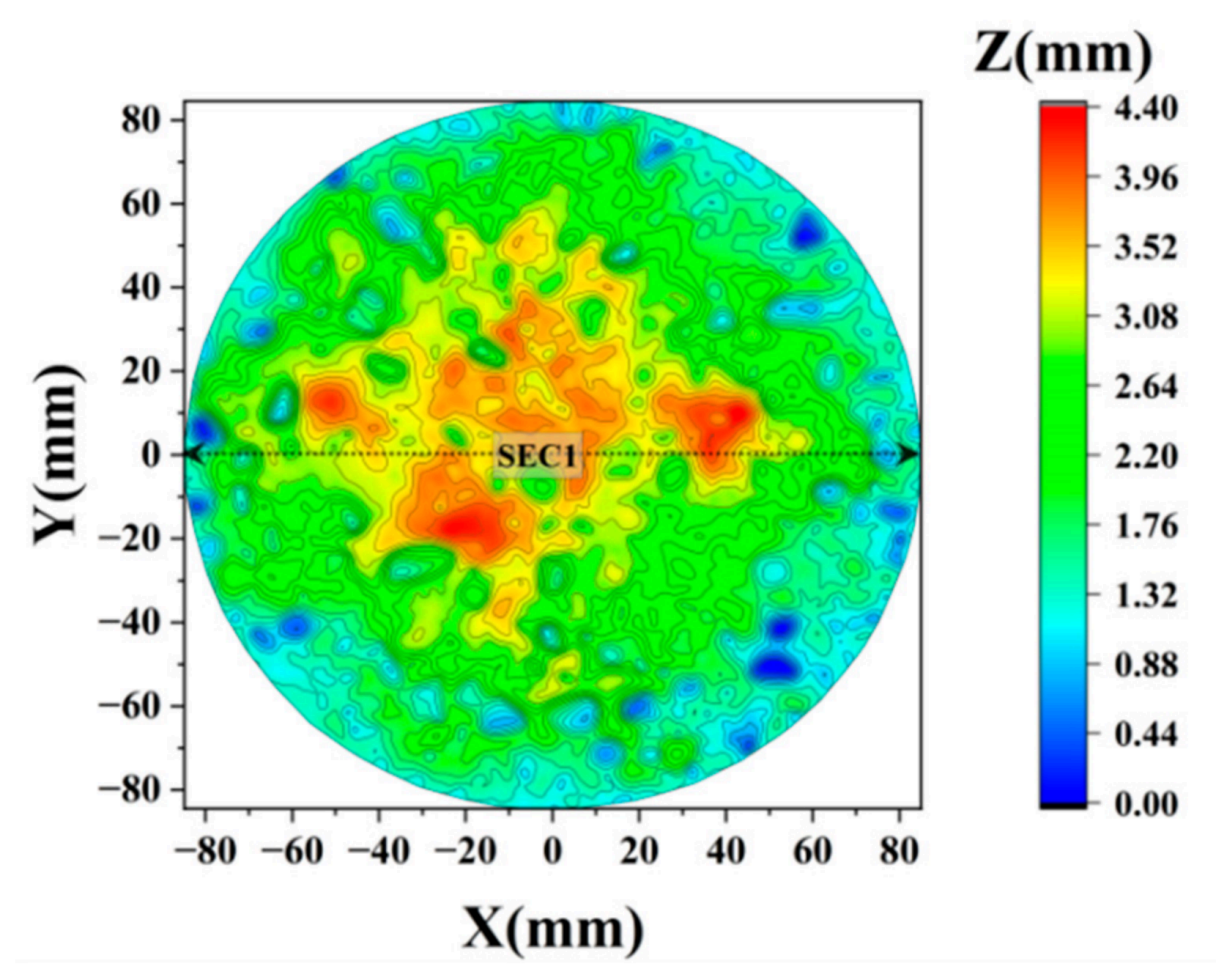
Vertical displacement contours of PVC-P GMB on JP4 cushion layer.

**Figure 22:**
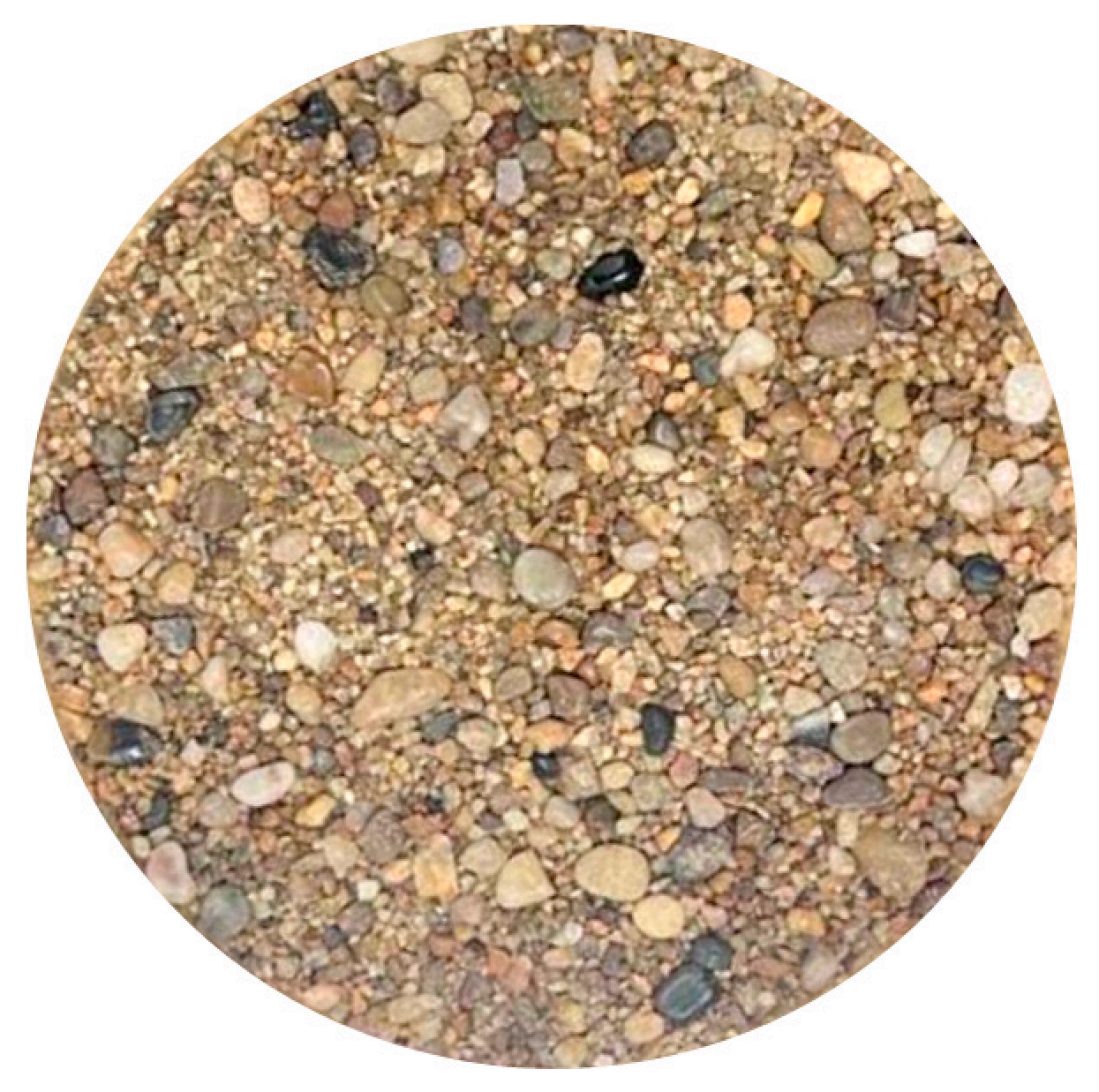
Layout of JP4 cushion layer.

**Figure 23:**
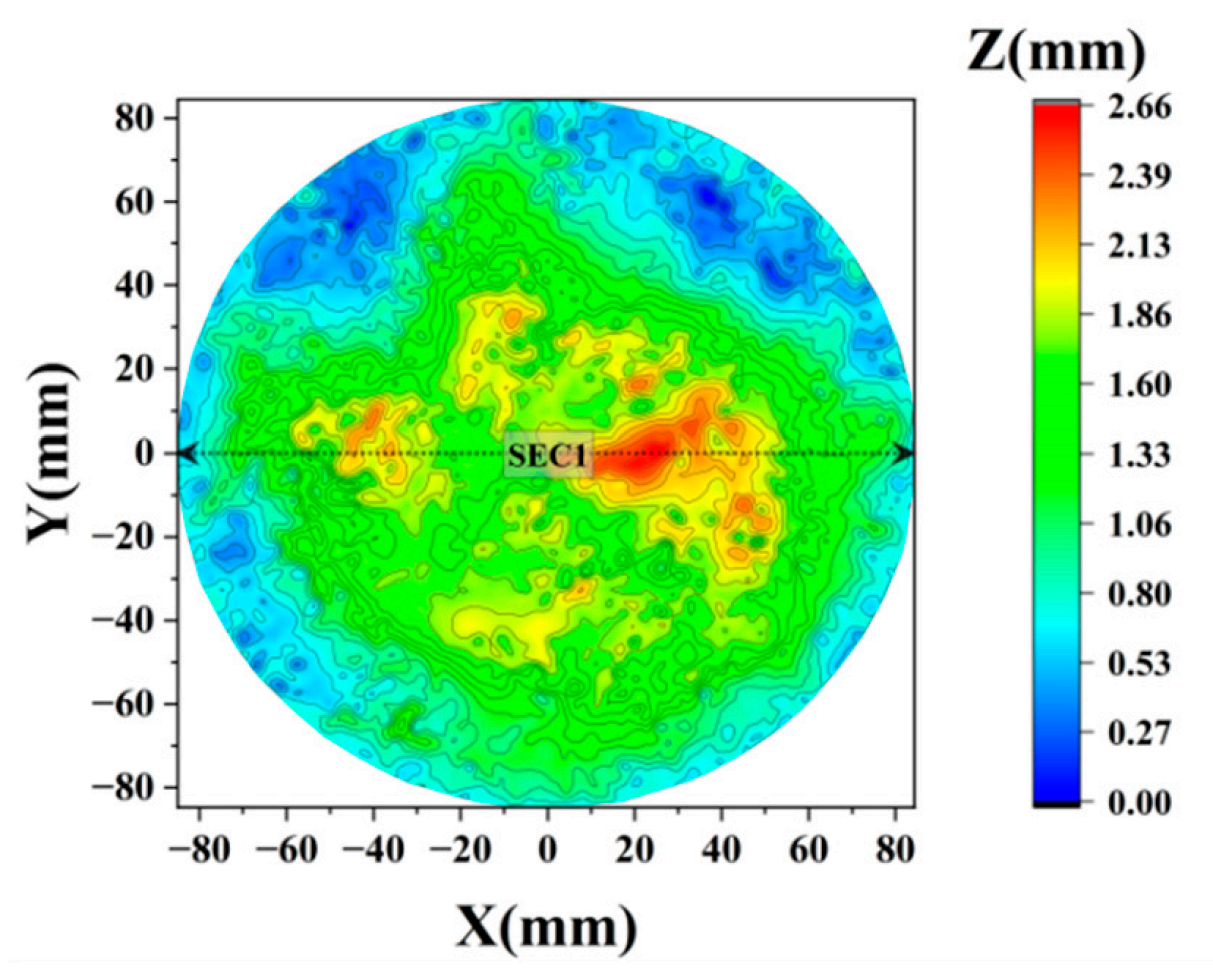
Vertical displacement contours of PVC-P GMB on JP5 cushion layer.

**Figure 24:**
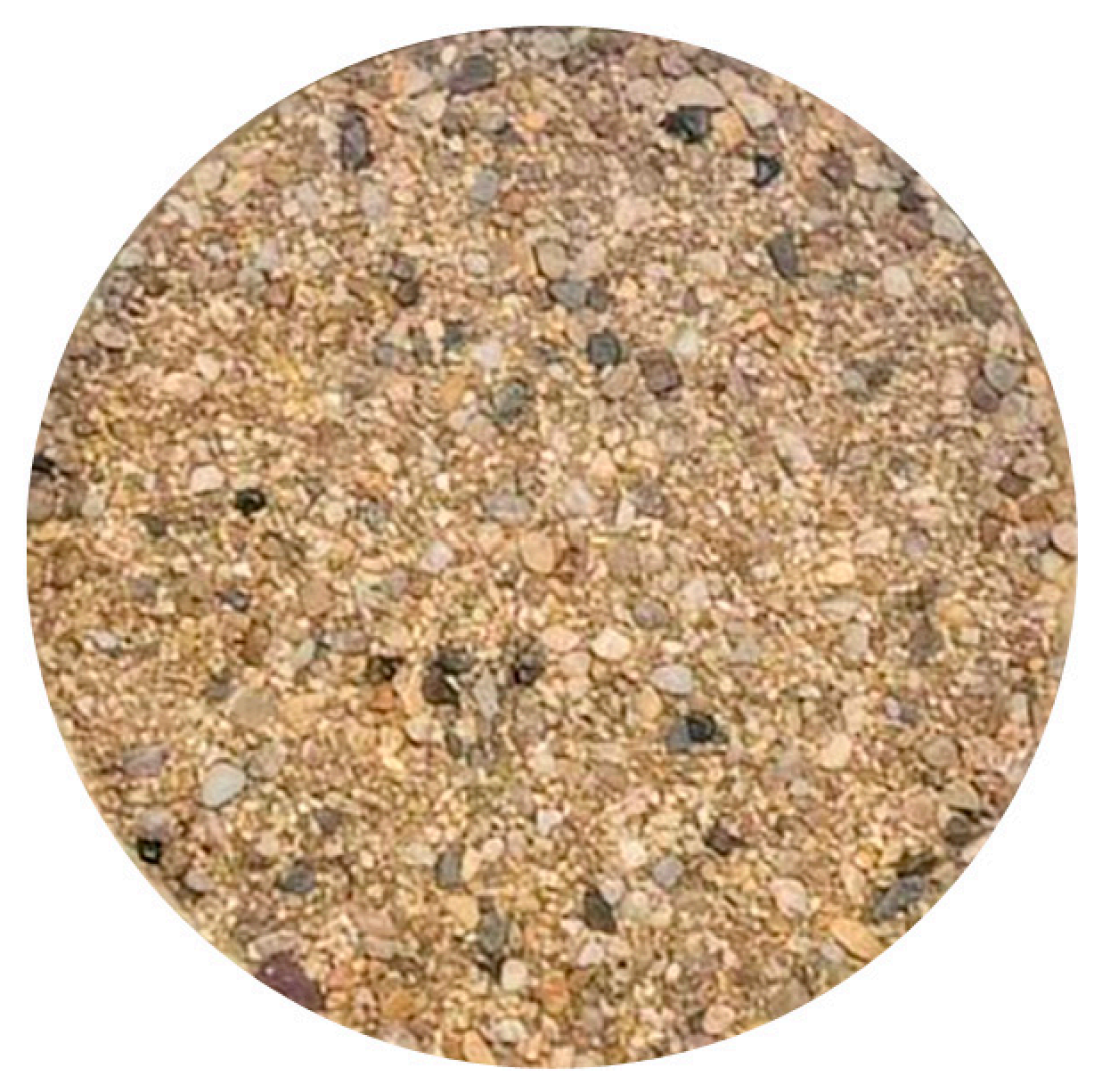
Layout of JP5 cushion layer.

Figure 15, Figure 16, Figure 17, Figure 18, Figure 19, Figure 20, Figure 21, Figure 22, Figure 23 and Figure 24 present the vertical displacement contours of PVC-P GMBs obtained from 3D laser scanning tests, together with the layout of cushion layers (JP1, JP2, JP3, JP4, and JP5). The vertical displacement of the PVC-P GMBs exhibited remarkable spatial heterogeneity. Accordingly, several representative high-displacement regions were defined for further analysis. For the JP1 cushion layer, multiple scattered high-displacement zones (ROI1 and ROI2) were observed, with a maximum vertical displacement up to 10.85 mm. Small displacements occurred in ROI3 and ROI4 regions. For JP2 cushion layer, larger vertical displacements appeared in ROI1, and the peak vertical displacement reached 10.10 mm. ROI2 and ROI3 regions exhibited relatively low displacements. These concentrated deformation regions corresponded to isolated large flat-rounded gravel particles, whereas the cushion layer with uniform and small particles yielded more uniform GMB deformation. In contrast, JP3 cushion exhibited distinct deformation behavior, with a continuous high-displacement zone (Figure 17), and the peak vertical displacement decreased significantly to merely 5.46 mm. JP4 and JP5 cushions followed similar deformation patterns to JP3; however, the maximum vertical displacements reduced to 4.40 mm and 2.66 mm, respectively. This is attributed to the fact that JP4 and JP5 cushions contain more fine particles, and inter-particle voids are sufficiently filled, leading to much flatter cushion surfaces and more uniform deformation distributions. Well-graded cushions with more fine particles yield moderate, distributed GMB deformation with low peak vertical displacement, thereby mitigating the local puncture risk of the GMB in pumped storage power stations.

**Figure 25:**
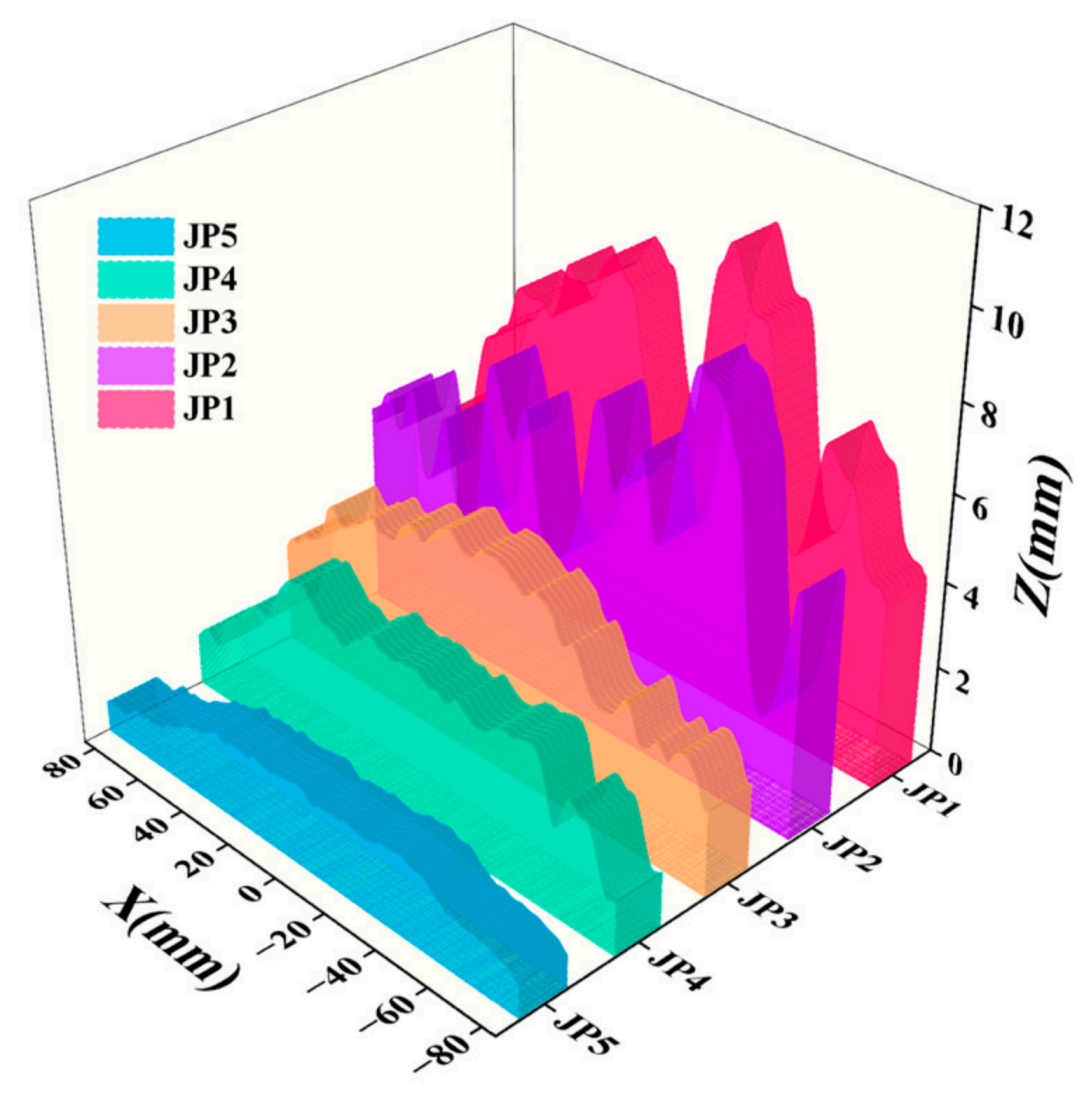
Vertical displacement of the PVC-P GMB on the horizontal profile for five cushions at 0.8 MPa.

Figure 25 plots the vertical displacement of PVC-P GMBs on the horizontal profile for five cushions at 0.8 MPa. It can be observed that JP5 cushion layer exhibited the lowest vertical displacement, whereas JP1 cushion layer displayed the highest value. As the maximum limiting particle size of the cushion decreased from 26.5 mm (JP1) to 4.75 mm (JP5), the maximum vertical displacement reduced from 10.85 mm to 2.66 mm. For JP5 and JP4 cushion layers, the vertical displacement exhibited a gentle curve with a low peak. In contrast, sharp and prominent peaks were observed in curves for JP2 and JP1. This is attributed to the large inter-particle voids and angular asperities of coarse particles within the cushion, which induce localized concentrated deformation in the GMB. The reduction in maximum limiting particle size lowered the surface roughness of the cushion in contact with the PVC-P GMB, thereby mitigating the GMB deformation under pressure. The results demonstrate that the cushion gradation exerts a considerable influence on the vertical displacement and subsequent strain developed in the GMB. Therefore, the effect of gradation parameters on the areal strain was explored in the following section.

#### 3.2.2. Areal Strain

**Figure 26:**
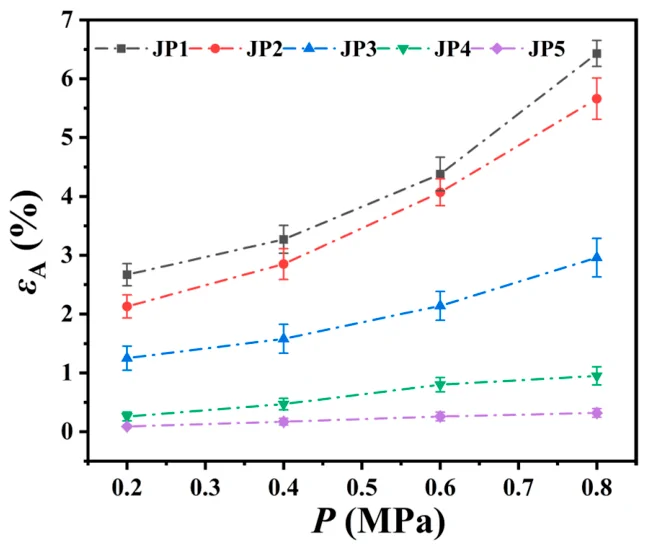
Relationship between areal strain and applied pressure for five flat-rounded gravel cushions.

The surface areas of the deformed lead sheets were acquired using a handheld SIMSCAN 3D laser scanner, and then the areal strain of PVC-P GMBs was calculated via Equation (8). Figure 26 reports the areal strains of PVC-P GMBs under various pressures for five flat-rounded gravel cushions (JP1–JP5). The areal strain of PVC-P GMBs increased monotonically with increasing applied pressure for all five gradation cushions. For the JP2 cushion, the areal strain increased from 2.13% to 5.66% with an increase in pressure from 0.2 MPa to 0.8 MPa. Considerable differences in areal strain were observed among different gradations. Under identical pressures, JP1 yielded the largest areal strain, followed by JP2, JP3 and JP4, while JP5 produced the minimum strain. At 0.8 MPa, the mean areal strain decreased remarkably from 6.43% (JP1) to 0.32% (JP5). Meanwhile, the standard deviation reduced from 0.22% to 0.07%, indicating that well-graded cushions not only mitigate local deformation, but also reduce the scatter induced by random void geometry. This is because the improvement in cushion gradation fills the large inter-particle voids, which significantly restrains the bulging deformation of the GMB and accordingly reduces the local areal strain.

Models were established linking the areal strain of PVC-P GMBs under five groups of flat-rounded gravel cushions to the width–depth ratio and the non-uniformity coefficient. Additional models were then developed to relate the width–depth ratio to the non-uniformity coefficient and the maximum limiting particle size.

**Figure 27:**
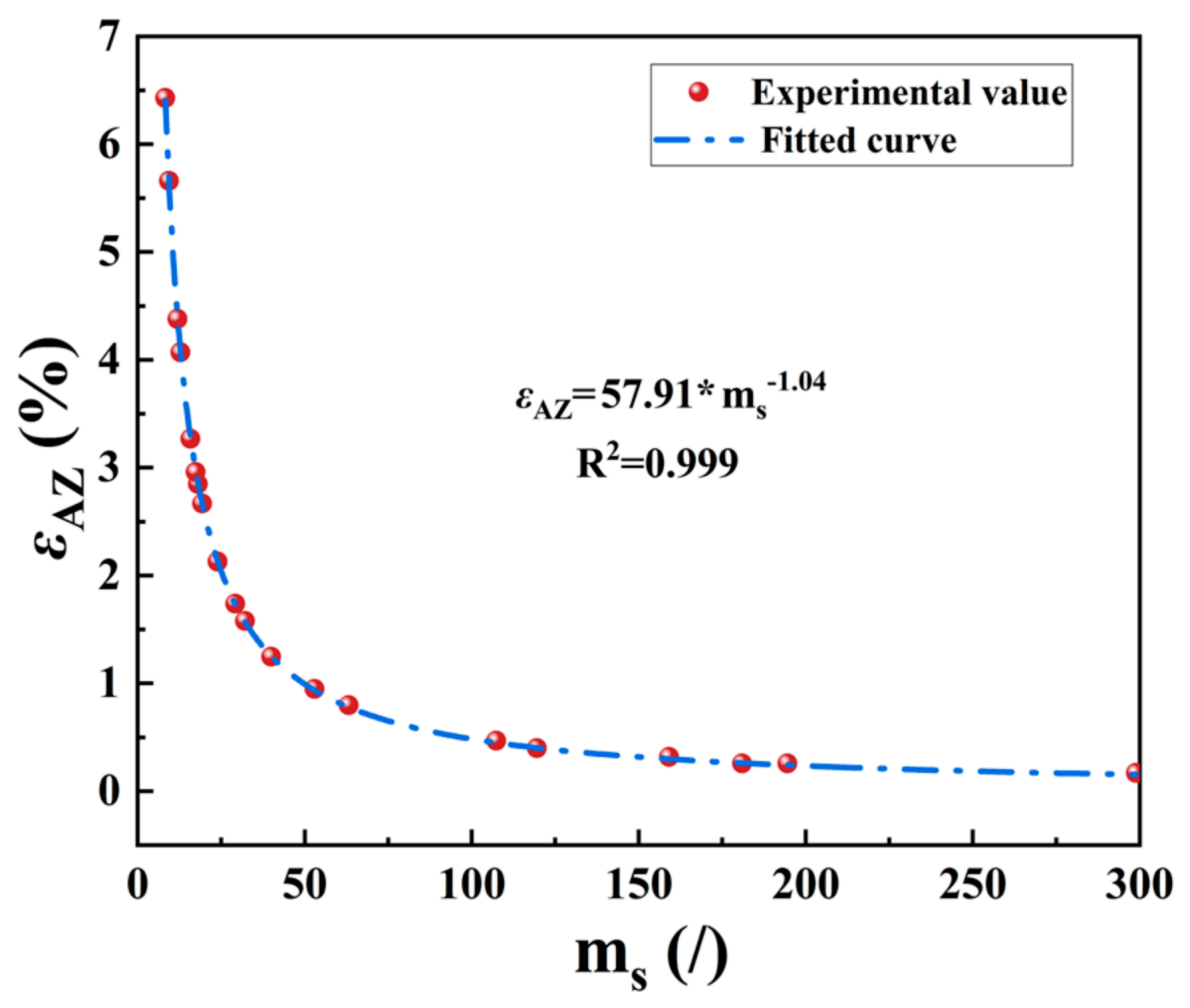
Relationship between areal strain and width–depth ratio.

Figure 27 presents the relationship between areal strain and width–depth ratio, and an allometric function was adopted to fit the areal strain with the width–depth ratio. A total of 20 data points were used for fitting. All fitted parameters were statistically significant (*p* < 0.05), with a determination coefficient of 0.999 and a RSS of 0.003, demonstrating good fitting performance. The function was expressed as follows:

(11) $$\varepsilon_{AZ}=57.91m_{s}^{-1.04}$$

The areal strain of the GMB on the flat-rounded gravel cushion showed a negative correlation with the width–depth ratio, which is consistent with the trend observed for the uniform particle cushion.

Likewise, the areal strain was fitted with the non-uniformity coefficient using an allometric function, and a high determination coefficient of 0.997 and a RSS of 0.08 were acquired. The function was given by:

(12) $$\varepsilon_{AZ}=1.72\times 10^{-14}C_{u}^{11.14}$$

**Figure 28:**
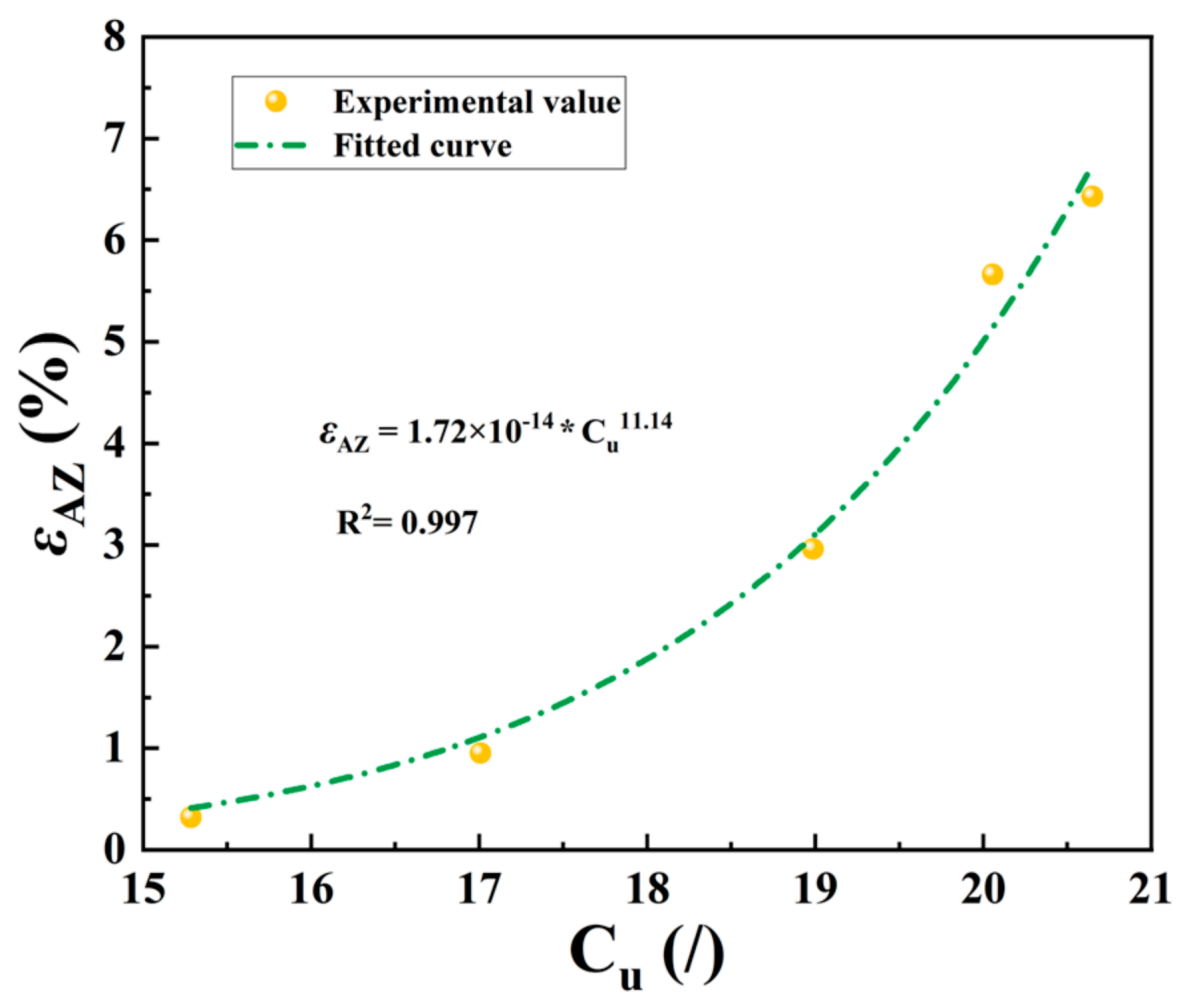
Empirical relationship between areal strain and non-uniformity coefficient.

Figure 28 depicts the empirical relationship between the areal strain and the non-uniformity coefficient. The areal strain of the GMB increased rapidly in a power law form with the increase in the non-uniformity coefficient. The strain increased mildly at low values, while a sharp rise in strain occurred with further increase in the non-uniformity coefficient. A larger non-uniformity coefficient implies greater particle size disparity within the cushion and a more poorly graded cushion, which can form local large inter-particle voids and induce local GMB bulging deformation, resulting in higher areal strain.

**Figure 29:**
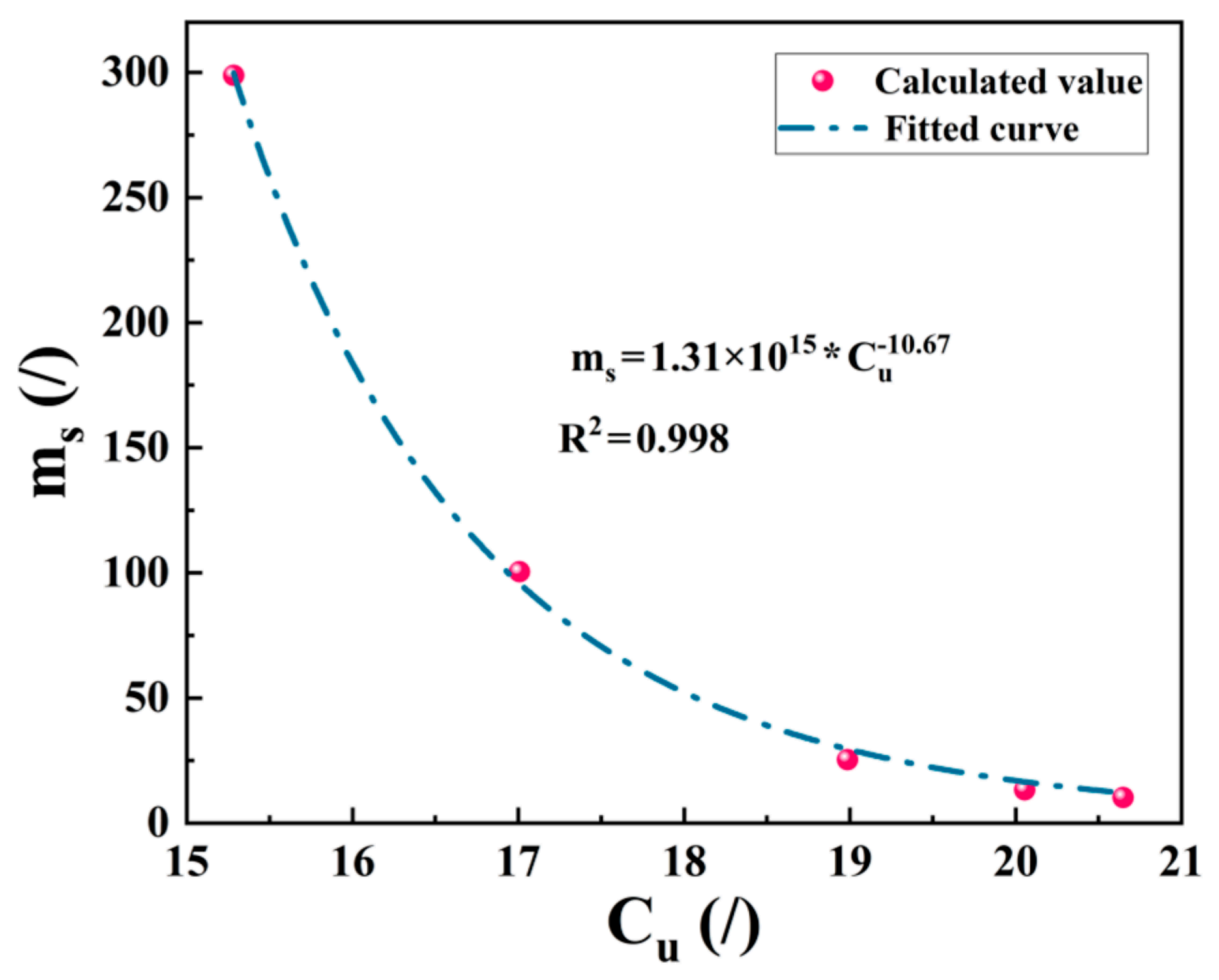
Empirical relationship between width–depth ratio and non-uniformity coefficient.

**Figure 30:**
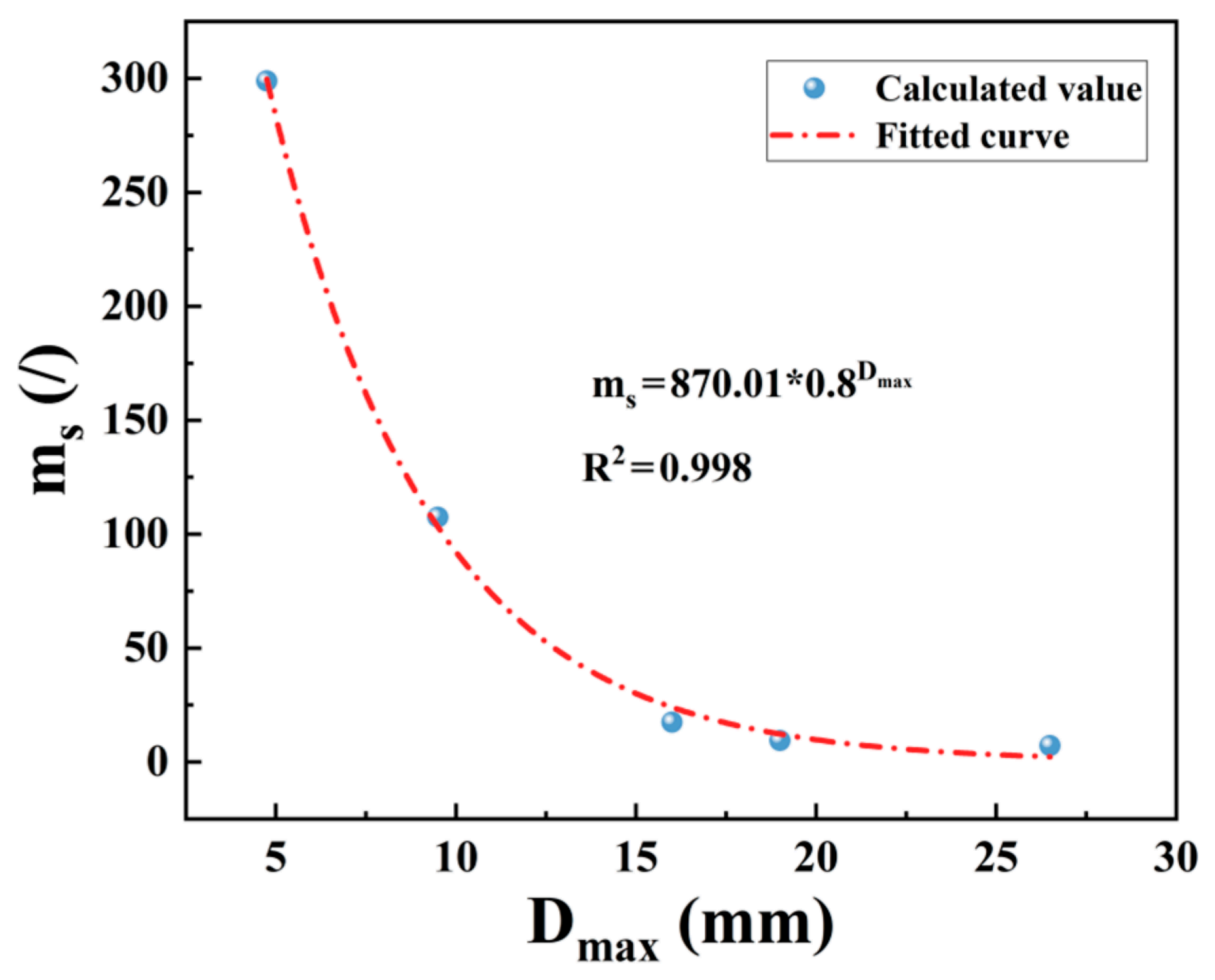
Empirical relationship between width–depth ratio and maximum limiting particle size.

Figure 29 and Figure 30 illustrate the empirical relationship between width–depth ratio and non-uniformity coefficient, as well as maximum limiting particle size. A pronounced allometric relationship exists between the width–depth ratio and non-uniformity coefficient, with a determination coefficient of 0.999, and the fitted equation is:

(13) $$m_{s}=1.31\times 10^{15}C_{u}^{-10.67}$$

Meanwhile, the empirical relationship between the width–depth ratio and maximum limiting particle diameter follows an Exp2P function, with a determination coefficient of 0.995. The fitted function is expressed as:

(14) $$m_{s}=870.01*0.8^{D_{max}}$$

The width–depth ratio decreased following a negative power law function with the increasing non-uniformity coefficient, and decayed exponentially as the maximum limiting particle size increased. At low values of the non-uniformity coefficient and maximum limiting particle size, the width–depth ratio presented high values. With increasing gradation heterogeneity and particle size, the width–depth ratio dropped sharply and gradually tended to level off. As a lower width–depth ratio induced larger areal strains, the non-uniformity coefficient and maximum limiting particle size should be controlled to satisfy the maximum allowable strain requirements. Under the present test scheme, as non-uniformity coefficient and maximum limiting particle size vary concurrently, the observed correlations may partly result from covariance between the gradation characteristics.

#### 3.2.3. Determination of Gradation Parameters

Tensile strains in GMBs develop at local indentations on underlying particles. Punctures occur in the GMB when the local tensile strains exceed the allowable values. The non-uniformity coefficient of flat-rounded gravel cushion should not be excessively large, as a larger non-uniformity coefficient can induce higher areal strain of the GMB. In addition, an increase in the maximum limiting particle size also increases the areal strain, which may endanger engineering safety. To ensure that the deformation of PVC-P GMBs in the reservoir basin of pumped storage power stations remains within a safe range, the maximum limiting particle size and non-uniformity coefficient should be rationally determined for the flat-rounded gravel cushion. According to the allowable biaxial tensile strain proposed by Shu [21] and Shu et al. [22], the allowable areal strain is taken as 20% of the strain corresponding to the peak stress, considering an adequate safety coefficient and the effects of long-term loading on the mechanical property degradation of GMBs during their service life. The PVC-P GMB on flat-rounded gravel cushions in this study is subjected to multi-axial tension, considering the long-term mechanical degradation and ensuring the GMB elastic deformation. The allowable areal strain is therefore defined as 20% of the peak strain in the elastic segment of the stress–strain curve acquired from liquid bulging tests.

**Figure 31:**
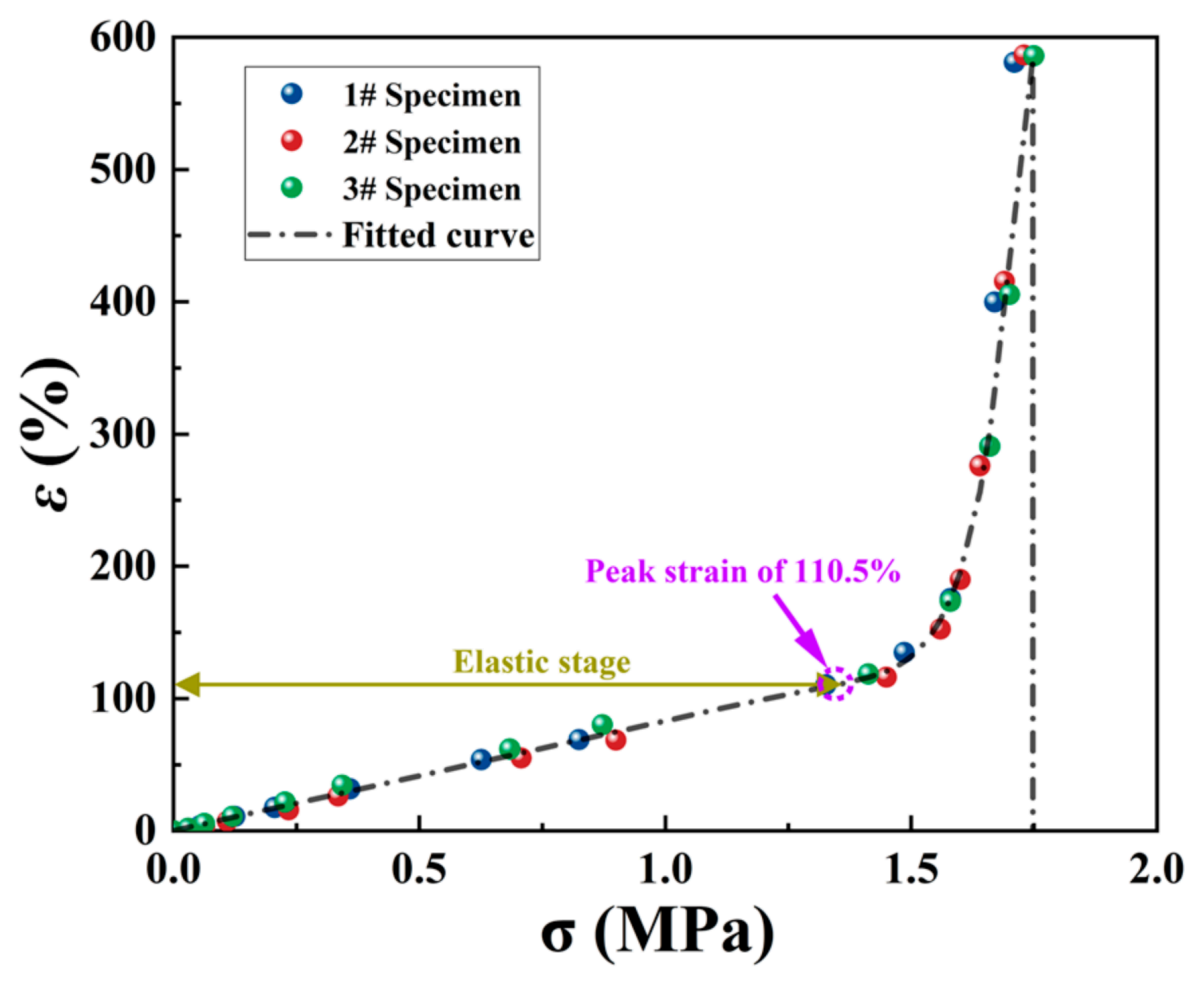
Stress–strain curve of PVC-P specimen in liquid bulging test.

Figure 31 presents the multi-axial tensile stress–strain curve of PVC-P GMB obtained from liquid bulging tests [23]. The peak strain in the elastic stage was 110.5%, accordingly, the allowable areal strain was determined to be 22.1%. Therefore, the areal strain of PVC-P GMB should satisfy:

(15) $$\varepsilon_{AZ}\leq {\left[\varepsilon \right]}_{AZ}=22.1\%$$

Equation (12) can be simplified as:

(16) $$C_{u}=e^{\frac{ln\frac{\varepsilon_{AZ}}{1.72\times 10^{-14}}}{11.14}}$$

Combining Equations (15) and (16), the non-uniformity coefficient was determined to be less than 22.71. Subsequently, the width–depth ratio was calculated as 4.42 using Equation (13). Finally, the maximum limiting particle size was obtained as 23.54 mm. Combined with the tested five gradations ($C_{u}$: 15.3–20.7, $D_{max}$: 4.75–26.5 mm), gradations satisfying the requirements correspond to a non-uniformity coefficient ranging from 15.3 to 20.7 and a maximum limiting particle size between 4.75 mm and 23.54 mm.

## 4. Conclusions

This study investigates the deformation behavior of PVC-P GMBs over uniform granular cushions and flat-rounded gravel cushions by employing a self-developed GMB deformation test apparatus and a 3D laser scanner. Based on the principle of equivalent circle, the correlations of linear strain and areal strain with the width–depth ratio were analyzed, and the gradation parameters satisfying the strain criterion for flat-rounded gravel cushions were determined. The main conclusions are summarized as follows:

1. Both the local linear strain and areal strain of PVC-P GMBs on uniform granular cushions follow an allometric function with the width–depth ratio, with determination coefficients higher than 0.994. For an identical width–depth ratio, the linear strain is always smaller than the areal strain. For engineering safety considerations, the areal strain is recommended to characterize the deformation of PVC-P GMBs on porous medium cushions.
2. A pronounced allometric relationship exists between the areal strain of PVC-P GMBs on flat-rounded gravel cushions and the width–depth ratio. The non-uniformity coefficient is negatively correlated with the width–depth ratio, and exhibits a positive correlation with the areal strain.
3. For the present laboratory conditions and flat-rounded gravel cushions, when the non-uniformity coefficient of flat-rounded gravel cushions ranges from 15.3 to 20.7, and the maximum limiting particle size is between 4.75 and 23.54 mm, the areal strain of the PVC-P GMBs can be constrained within 20% of the peak elastic strain. This ensures that the PVC-P GMB remains in the elastic deformation stage under the investigated laboratory conditions.

This paper investigates the strain characteristics of PVC-P GMBs on granular cushions, and obtains the gradation parameters satisfying the deformation requirements for flat-rounded gravels. The findings contribute to a deeper understanding of the local deformation behavior of PVC-P GMBs on cushion layers, providing technical support for cushion design in pumped storage reservoir basins using GMBs as anti-seepage materials. However, due to the discrepancy in mechanical properties between lead sheets and PVC-P GMBs, deformed lead sheets cannot truly reflect the actual deformation of the GMB. Therefore, advanced equipment and algorithms are recommended to directly investigate the strain behavior of PVC-P GMBs in future studies. Furthermore, as the drainage performance of the cushion layer is not evaluated in this study, further experimental investigations are required to obtain the optimal gradation satisfying drainage and protection performance in practical engineering.

## Figures and Tables

**Table 1:** Index properties of tested PVC-P GMBs.

Property	Method	Horizontal	Longitudinal	Unit
Thickness	ASTM D5199-25, 2025 [17]	1.5	1.5	mm
Mass per unit area	ASTM D5261-10, 2024 [18]	0.22	0.22	g/cm^2^
Break strength	ASTM D638-22, 2022 [19]	159.45	157.52	MPa
Yield strength	ASTM D638-22, 2022 [19]	14.62	12.07	MPa
Break elongation	ASTM D638-22, 2022 [19]	135.88	133.54	%
Yield elongation	ASTM D638-22, 2022 [19]	59.39	53.02	%

**Table 2:** Physical and mechanical properties of lead sheet.

Type	Purity (%)	Tensile Strength (MPa)	Yield Strength (MPa)	Break Strain (%)	Hardness (HBS)
Lead sheet	99.999	17.2	7.4	80.3	4.1

**Table 3:** Parameters of transparent glass spheres.

Type	Density (g/cm^3^)	Degree of Roundness (%)	Elastic Modulus (GPa)	Surface Friction Coefficient	Knoop Hardness (HK)	Rockwell Hardness (HRC)	Vickers Hardness (HV)
Glass spheres	2.45	99.6	116.4	0.20	515	48	540

**Table 4:** Gradation parameters of flat-rounded gravel cushion.

Cushion Layer	Non-Uniformity Coefficient *C_u_*	Coefficient of Curvature *C_c_*	Maximum Limiting Particle Size *D_max_* (mm)	Pressure *P* (MPa)			
JP1	20.65	2.10	26.50	0.2	0.4	0.6	0.8
JP2	20.06	1.78	19.00	0.2	0.4	0.6	0.8
JP3	18.99	2.19	16.00	0.2	0.4	0.6	0.8
JP4	17.01	2.07	9.50	0.2	0.4	0.6	0.8
JP5	15.28	2.11	4.75	0.2	0.4	0.6	0.8

**Table 5:** Technical parameters of the handheld 3D laser scanner.

Item	Value
Scanning Mode	High-speed Scanning: 34 crossed blue laser beams
	Fine Scanning: 7 parallel blue laser beams
	Deep Hole Scanning: Additional 1 blue laser beam
Maximum Accuracy	0.020 mm
Maximum Scanning Rate	1,250,000 measurements/s
Scanning Field of View	650 mm × 550 mm
Maximum Resolution	0.020 mm
Maximum Volumetric Accuracy	0.015 mm + 0.035 mm/m
Working Distance	300 mm
Depth of Field	550 mm
Dimensions	203 mm × 80 mm × 44 mm
Weight	570 g

## Data Availability

The original contributions presented in this study are included in the article. Further inquiries can be directed toward the corresponding author.

## Abbreviations

The following abbreviations are used in this manuscript:

PVC-P	Plasticized polyvinyl chloride
GMB	Geomembrane
HDPE	High-density polyethylene
3D	Three-dimensional
RSS	Residual sum of squares
